# New Insights into the Mechanism of Antibacterial Action of Synthetic Peptide *Mo*-CBP_3_-PepI against *Klebsiella pneumoniae*

**DOI:** 10.3390/antibiotics11121753

**Published:** 2022-12-04

**Authors:** Levi A. C. Branco, Pedro F. N. Souza, Nilton A. S. Neto, Tawanny K. B. Aguiar, Ayrles F. B. Silva, Rômulo F. Carneiro, Celso S. Nagano, Felipe P. Mesquita, Luina B. Lima, Cleverson D. T. Freitas

**Affiliations:** 1 Department of Biochemistry and Molecular Biology, Federal University of Ceará, Fortaleza 60020-181, CE, Brazil; 2 Drug Research and Development Center, Department of Physiology and Pharmacology, Federal University of Ceará, Fortaleza 60020-181, CE, Brazil; 3 Department of Fisheries Engineering, Federal University of Ceará, Fortaleza 60020-181, CE, Brazil

**Keywords:** multidrug-resistant bacteria, proteomic analysis, synthetic peptides, antibacterial peptides

## Abstract

*Klebsiella pneumoniae* is a multidrug-resistant opportunistic human pathogen related to various infections. As such, synthetic peptides have emerged as potential alternative molecules. *Mo*-CBP_3_-PepI has presented great activity against *K. pneumoniae* by presenting an MIC_50_ at a very low concentration (31.25 µg mL^−1^). Here, fluorescence microscopy and proteomic analysis revealed the alteration in cell membrane permeability, ROS overproduction, and protein profile of *K. pneumoniae* cells treated with *Mo*-CBP_3_-PepI. *Mo*-CBP_3_-PepI led to ROS overaccumulation and membrane pore formation in *K. pneumoniae* cells. Furthermore, the proteomic analysis highlighted changes in essential metabolic pathways. For example, after treatment of *K. pneumoniae* cells with *Mo*-CBP_3_-PepI, a reduction in the abundance of protein related to DNA and protein metabolism, cytoskeleton and cell wall organization, redox metabolism, regulation factors, ribosomal proteins, and resistance to antibiotics was seen. The reduction in proteins involved in vital processes for cell life, such as DNA repair, cell wall turnover, and protein turnover, results in the accumulation of ROS, driving the cell to death. Our findings indicated that *Mo*-CBP_3_-PepI might have mechanisms of action against *K. pneumoniae* cells, mitigating the development of resistance and thus being a potent molecule to be employed in producing new drugs against *K. pneumoniae* infections.

## 1. Introduction

The emergence of multidrug-resistant bacteria (MDRB) is a challenge to public health worldwide, leading to lengthy hospital stays, high healthcare costs around USD 1 million, and a death rate of 46.31% [1,2,3]. *K. pnuemoniae* is a Gram-negative bacterium that colonizes the human gastrointestinal system and often is found in feces [4]. Additionally, *K. pneumoniae* is a bacterium belonging to the ESKAPE (*Enterococcus faecium*, *Staphylococcus aureus*, *Klebsiella pneumoniae*, *Acinetobacter baumannii*, *Pseudomonas aeruginosa*, and *Enterobacter* species) group of MDRB that are pathogenic to humans [5,6].

*K. pneumoniae* is an opportunistic MDRB only able to infect people with a compromised immune system [4]. *K. pneumoniae* presents resistance against polymyxins, carbapenems, fluoroquinolones, aminoglycosides, tetracyclines, third-generation cephalosporins, and is pan drug-resistant [7,8,9]. The clinical manifestations of *K. pneumoniae* present as an acute bacterial skin infection, bacteremia, pneumonia, and osteoarticular infection [10,11,12]. 

The multidrug resistance acquired by *K. pneumonia* made this bacterium an important pathogen threatening human health. Based on that, it is emergent and imperative to seek new molecules to fight back in two ways: (1) to develop a new drug to produce a new treatment; or (2) to produce a new molecule that could act synergistically with commercial drugs making them effective again [1]. In this scenario, antimicrobial peptides represent a notorious group of molecules that could help scientists quickly find a way to cope with antimicrobial resistance [13]. However, natural antimicrobial peptides have presented problems in clinical trials, such as toxicity to host cells, low resistance to proteolysis, and sometimes high cost of obtention [13,14].

Synthetic antimicrobial peptides emerged as a solution to solve the problems presented by natural antimicrobial peptides. Synthetic antimicrobial peptides are designed using a natural model sequence to have higher antimicrobial activity, greater resistance to proteolysis, and no toxicity to host cells. Recently, our research group has designed synthetic peptides called *Mo*-CBP_3_-PepI using the sequence of a chitin-binding protein from *Moringa oleifera* [15]. *Mo*-CBP_3_-PepI is a non-hemolytic small cationic peptide with a net charge +1, a molecular mass of 893.10 Da, and a hydrophobic ratio of 62%. It has a secondary structure as α-helix confirmed by circular dichroism assays [15]. 

Regarding the antimicrobial potential, *Mo*-CBP_3_-PepI presents great anticandidal activity against *Candida albicans*, *C. parapsilosis*, *C. krusei*, and *C. tropicalis*. The mechanisms of action of *Mo*-CBP3-PepI against the *Candida* genus were revealed using *C. albicans* as a model [15,16,17]. Regarding the antibacterial activity, *Mo*-CBP3-PepI, the only relevant activity, was evaluated against *K. pneumoniae*. Therefore, this study employed fluorescence and scanning electron microscopes and proteomic analysis to provide new insights into the mechanism of antibacterial action of *Mo*-CBP_3_-PepI against *K. pneumoniae*. Additionally, toxicity tests were performed against human cells to produce new data on the safety of *Mo*-CBP_3_-PepI. 

## 2. Material and Methods

### 2.1. Biological Material

The human-pathogenic Gram-negative bacteria *K. pneumoniae* (ATCC 10031) strain was obtained from the Laboratory of Plant Toxins (LABTOX) of the Federal University of Ceará (UFC).

### 2.2. Peptide Synthesis

The synthetic peptide *Mo*-CBP_3_-PepI (CPAIQRCC) [15] was chemically synthesized by the company ChemPeptide (Shanghai, China), where its purity and quality of it were tested by mass spectrometry and reverse-phase high-performance liquid chromatography (Figure S1 (Supplementary Materials)).

### 2.3. Cell Viability by MTT Assay

The cell viability assay was performed as described by Lima et al. [17], with some adjustments. After the antibacterial assay was completed as described by Oliveira et al. [15], the wells containing the treated cells with *Mo*-CBP_3_-PepI at 1 mg mL^−1^ and control cells (50 µL) were incubated for 3 h in the dark at 37 °C with 50 µL of 3-(4,5-dimethylthiazol-2-yl)-2,5-diphenyltetrazolium bromide (2 mg mL^−1^, MTT). After incubation, 100 µL of 100% DMSO was added to the wells, and the plate was slowly shaken to dissolve the formazan crystals. The absorbance was measured using a microplate reader (Epoch, BioTek) with a wavelength of 495 nm. The controls used for this assay were 5% of dimethyl sulfoxide (DMSO) prepared in 0.15 M NaCl (saline) solution and ciprofloxacin (1000 µg mL^−1^) prepared in 5% ethanol in sterile saline solution.

### 2.4. Antibiofilm Assay

The antibiofilm assay was conducted in flat-bottom 96-well polystyrene microplates as described by Neto et al. [18]. A single colony of *K. pneumoniae* was collected in stock Petri plates containing inoculated Mueller–Hinton agar. The colony was used as inoculum for 5 mL of Mueller–Hinton broth, and the media was incubated for 24 h in the dark at 37 °C. In sequence, the O.D. of the cell suspension was measured using a microplate reader (Epoch, BioTek) and adjusted to 0.1 at 630 nm with Mueller–Hinton broth. For the inhibition of biofilm formation, 50 µL of the cell (2.5 × 10^−5^ CFU mL^−1^) suspension was incubated with 50 µL of the peptide (31.25 μg mL^−1^) solution and 50 µL of the respective controls described above. The incubation lasted for 48 h at 37 °C. For the biofilm degradation assay, 50 µL of the cell suspension was incubated for 24 h at 37 °C. After this, 50 µL of the peptide solution and the respective controls were added to the wells containing the preformed biofilm and incubated for 24 h at 37 °C. 

After incubation, both wells of biofilm formation inhibition and biofilm degradation were washed once with a sterile saline solution. Then, the wells were fixated with 100 µL methanol 99% for 15 min. After the methanol was removed and the plates were dried at 37 °C, then, the wells were stained with 200 µL of 0.1% violet crystal solution for 20 min. In sequence, the wells were washed three times with distilled water. Then, the crystals stained on the biofilm were diluted with 200 µL of 33% acetic acid, and the O.D was measured using a wavelength of 600 nm. The assay was executed in triplicate, with three independent biological experiments.

### 2.5. Mechanism of Action Evaluation by Fluorescence Microscopy

#### 2.5.1. Cell Membrane Integrity by Propidium Iodide (PI) and FITC-Dextran Uptake

The assay was conducted as described by Oliveira et al. [15], with some modifications. Preparation of cell suspension was completed as mentioned above. After, 50 µL of the diluted bacteria solution was incubated in the dark for 24 h, at 37 °C, with 50 µL of the peptide solution (31.25 µg mL^−1^) previously prepared with 5% of dimethyl sulfoxide (DMSO) diluted in 0.15 M NaCl solution. The assay was conducted in 1.5 mL microtubes. The diluted bacteria solution was incubated only with the DMSO-NaCl solution for control. After incubation, the microtubes were centrifuged (5000× *g*, 5 min, 4 °C) and washed three times. Next, the washed cells were incubated with 1 µM propidium iodide for 30 min, in the dark, at 37 °C. After this, the cells were washed two times with saline solution to remove the excess fluorophore and observed with a fluorescence microscope (Olympus System BX 60; excitation wavelength, 488 nm; emission wavelength, 525 nm).

Additionally, to know the size of the pore formed, in a new experiment completed precisely as above, cells were incubated with FITC-Dextran (fluorescein isothiocyanate (FITC)-Dextran) with a size of 10-kDa. After incubation for 30 min, cells were washed with 0.15 M of NaCl and visualized in light and fluorescence microscope Olympus System BX 60 with an excitation wavelength of 490 nm and emission wavelength of 520 nm. 

#### 2.5.2. Detection of Peptide-Induced Overproduction of Reactive Oxygen Species (ROS)

This assay was conducted in the same way as above but with a few differences. The preparation of cell suspension and the antimicrobial assay was performed in the same way described above. After the three rounds of centrifugation and washes, the cells were incubated with 1 mM 2,7-dichlorofluorescein diacetate (DCFH-DA) [19] for 30 min, in the dark, at 37 °C. After the washes, the cells were also observed under a fluorescence microscope (Olympus System BX60; excitation wavelength, 488 nm; emission wavelength, 525 nm).

#### 2.5.3. Scanning Electronic Microscopy (SEM) Analysis

SEM analysis was conducted as described by Staniszewska et al. [20]. After the antibacterial assay, cells were centrifuged (5000× *g*, 5 min, 4 °C), the supernatant was removed, cells resuspended, and fixated for 5 h in a fixation solution (2.5% glutaraldehyde (*v*/*v*) in 0.15 M Na-phosphate buffer, pH: 7.2). After centrifugations as described above, the cells were washed three times with 0.15 M Na-phosphate buffer, pH: 7.2. For dehydration, the samples were incubated and dried with ethanol (30%, 50%, 70%, 100%, 100% (*v*/*v*)) for 10 min each, and centrifugation as described above after each incubation time. Lastly, the samples were incubated with 50/50 ethanol/hexamethyldisilane (HMDS) for 10 min and centrifuged. Then, the pellet was washed with 100% HMDS and transferred to a coverslip to dry out. After complete drying, the coverslips were assembled on stubs and coated with a 20 nm gold layer using a PET coating machine (EMITECH—Q150TES; Quorum Technologies, Lewes, England). The SEM analyses were completed with an Inspect™ 50 FEI Scanning Electron Microscope, equipped with a low energy detector (Everhart Thornley detector), using an acceleration beam voltage of 20,000 kV and 20,000× detector magnification.

### 2.6. Protein Extraction and Gel-Free Proteomic Analysis

Initially, an antibacterial assay was performed within 24 h of incubation, using the best inhibitory concentration of *Mo*-CBP_3_-PepI (31.25 µg mL^−1^) [15]. After this, samples were washed twice with 50 mM sodium acetate pH 5.2, with centrifugations at 12,000× *g* for 15 min at 4 °C. At the end of the washes, the samples were resuspended in 300 µL in the same buffer and frozen for 24 h. Then, the frozen samples were sonicated for 30 min to break the cell wall and membrane, the samples were centrifuged again, and the supernatant was collected.

After that, the Bradford assay was performed to determine the protein concentration in the samples. This step was followed by adding a 10 mM DTT solution under incubation for 1 h at 37 °C to reduce the proteins. Then iodoacetamide was added to a final concentration of 15 mM and incubated for 30 min in a dark room to alkylate the reduced proteins. The proteins reduced and alkylated were digested using trypsin gold (Promega, Madison, WI, USA) to a final concentration of 1:20 (*w*/*w*) as described by manufacturers. The trypsin digestion was performed for 16 h at 37 °C. Finally, the samples were dried in a speed vacuum (Eppendorf, Hamburg, Germany) for 3 h and analyzed by ESI-QUAD-TOF mass spectrometer.

### 2.7. Protein Identification

Tandem mass spectra were extracted into PKL files for both samples, and the proteins were searched using MASCOT MS/MS ions search from MATRIX SCIENCE (https://www.matrixscience.com/cgi/search_form.pl?FORMVER=2&SEARCH=MIS (accessed on 15 September 2022)) against UP625_E_coli_K12 (AA), UP808_K_pneumoniae and SwissProt databases (the taxonomy was set in bacteria). The terms for the search were: fixed modifications to Carbamidomethyl (C); variable modifications to Oxidation (O); the Peptide tolerance was set to 1.2 DA (with 1% FDR); the MS/MS tolerance was set to 0.6 DA; the peptide charge was set to 2+, 3+, and 4+; and finally, the instrument was set to ESI-QUAD-TOF. The proteins identified in both samples were searched for in UNIPROT and separated into 3 sets (unique from control, unique from the cells treated with *Mo*-CBP_3_-PepI, and *Mo*-CBP_3_-PepI x control shared proteins). 

The proteins with a fold-change value ≥ 1.5 (*p* < 0.05, Tukey’s test) that were up-accumulated (increased the abundance), and proteins with a fold-change value ≤ 0.5 (*p* < 0.05, Tukey’s test) that were down-accumulated (decreased the abundance) were taken into consideration for comparisons. For each protein, its corresponding FASTA file was downloaded. Then, the blast2go program (https://www.blast2go.com/ (accessed on 30 September 2022)) was used to categorize the proteins detected by Gene Ontology (GO) annotation according to Molecular function, Biological Activity, and subcellular location.

### 2.8. Cytotoxicity Assay

The cytotoxicity assay was assessed by measuring the ability of live cells to convert a yellow dye, 3-(4,5-dimethyl-2-thiozolyl)-2,5-diphenyl-2H-tetrazolium bromide (MTT), to formazan. The cell lines used were L929 (murine fibroblasts, ATCC number CCL-1), MRC-5 (human lung fibroblasts, ATCC number CCL-171), and HaCaT (human keratinocytes) following the methodology described by Souza et al. [14]. Cells were treated with *Mo*-CBP_3_-PepI at 1 mg mL^−1^. The alkylating agent methyl methanesulfonate (MMS) at 4 × 10^−5^ M was used as the positive control. The MTT formazan product was dissolved in 150 µL of DMSO, and the absorbance was measured using a multiplate reader (Spectra Count, Packard, Mississauga, ON, Canada). The drug effect was quantified as the percentage of control absorbance of the reduced dye at 595 nm.

### 2.9. Comet Assay

For this assay, MMS at 4 × 10^−5^ M was used as the positive control for DNA damage, and peptides were assayed at 1 mg mL^−1^. The standard alkaline comet assay (single-cell gel electrophoresis) after treatment (24 h). Cells were washed with ice-cold PBS, trypsinized, and resuspended in a complete medium. Thus, 20 µL of cell suspension (0.7 × 10^5^ cells/mL) was dissolved in 0.75% low-melting-point agarose. Slides were incubated in ice-cold lysis solution (2.5 M NaCl, 0.01 M Tris, 0.1 M EDTA, 1% Triton X-100, and 10% DMSO, pH 10.0) at 4 °C for at least 1 h to remove cell membranes, leaving DNA as “nucleoids”. Afterward, the slides were placed in a horizontal electrophoresis unit and incubated with fresh buffer solution (0.3 M NaOH, 0.001 M EDTA, pH 13.0) at 4 °C for 20 min to allow DNA to unwind and the expression of alkali-labile sites. Electrophoresis was conducted for 20 min at 25 V (94 V/cm). All the above steps were performed in the dark to prevent additional DNA damage. Slides were neutralized (0.4 M Tris, pH 7.5) and stained using 20 µg mL of ethidium bromide. Three hundred cells (100 cells from each of the three replicate slides for each treatment) were selected, coded, and blindly analyzed for DNA migration. These cells were visually scored according to tail length into five classes:

Class 0: undamaged, without a tail;
Class 1: with a tail shorter than the diameter of the head nucleus;
Class 2: with tail length 1–2× the diameter of the head;
Class 3: with a tail longer than 2× the diameter of the head;
Class 4: comets with no heads.

The index of damage (ID) value was calculated for each sample. The ID was defined as an arbitrary score based on the number of cells in the different damage classes, which are visually scored by measuring DNA migration length and the amount of DNA in the tail. DI ranges from 0 (no tail: 100 cells × 0) to 400 (with maximum migration: 100 cells × 4).

### 2.10. Morphological Analysis of Apoptotic Cells

For this assay, the concentration of peptides was 1 mg mL^−1^. DMSO-NaCl was the negative control for damage, and MMS at 4 × 10^−5^ M was used as the positive control for DNA damage. Peptides and control solutions were incubated as described above. Cells with the morphological characteristics of apoptosis, such as apoptotic bodies, peripheral condensation of chromatin, fragmented nucleus, and small cell volume, were evaluated after 24 h of treatment, either with controls or peptides. The evaluation was executed using acridine orange (AO)/ethidium bromide (EB) staining assay. A total of 50 µL of the cell suspension was mixed with 1 µL of the staining solution (100 µg/mL AO + 100 µg/mL EB in PBS) and spread on a slide, where 300 cells were counted per data point.

## 3. Results and Discussion

### 3.1. Cell Viability and Antibiofilm Activity of Mo-CBP3-PepI against K. pneumoniae

As reported in a previous work by Oliveira et al. [15], *Mo*-CBP_3_-PepI presented an MIC_50_ against *K. pneumoniae* at 31.25 µg mL^−1^. The experiments we repeated here led to the same results, corroborating the data presented by Oliveira et al. [15]. The concentration of *Mo*-CBP_3_-PepI to reach an MIC_50_ against *K. pneumoniae* is very low compared to other synthetic peptides [21,22]. For example, Fleeman et al. [21] showed that the synthetic peptide called PepC, which presented an MIC_50_ at a concentration of 350 µg mL^−1^ 11 times higher than the concentration presented by *Mo*-CBP_3_-PepI. Additionally, Tincho et al. [22] tested three synthetic peptides, and all presented an MIC_50_ against *K. pneumoniae* at a concentration of 500 µg mL^−1^, 16.07 times higher than *Mo*-CBP_3_-PepI. These results revealed that *Mo*-CBP_3_-PepI is much more effective against *K. pneumoniae* than other synthetic peptides. 

We further evaluated the cell viability of *K. pneumoniae* cells and biofilm formation after treatment with *Mo*-CBP_3_-PepI. The first was to evaluate the number of viable cells after treatment with *Mo*-CBP_3_-PepI (Table 1). The MTT assay revealed that only 47.54% ± 0.008 of *K. pneumoniae* cells were viable after incubation with *Mo*-CBP_3_-PepI, which agrees with the data of MIC_50_. These results prove that *Mo*-CBP_3_-PepI kills half of the cells at the tested concentration. Otherwise, 100% of *K. pneumoniae* cells were viable in control treated with 5% DMSO in 0.15 M NaCl (Table 1).

Moving forward, the antibiofilm potential of *Mo*-CBP_3_-PepI toward *K. pneumoniae* was evaluated. *Mo*-CBP_3_-PepI barely inhibited the biofilm formation of *K. pneumoniae*, only 11.87% ± 0.001, and presented no activity against the preformed biofilm (Table 1). As expected, the control solution was ineffective in inhibiting the formation or degrading the preformed biofilms of *K. pneumoniae* (Table 1).

### 3.2. Toxicity of Mo-CBP_3_-PepI to Human Cells

Before we move forward with the study to understand the mechanisms of action of *Mo*-CBP_3_-PepI against *K. pneumoniae,* toxicity tests against human cells were needed to provide information that might indicate the application of *Mo*-CBP_3_-PepI. For example, Oliveira et al. [15] presented a safe *Mo*-CBP_3_-PepI based on the absence of hemolytic activity against human red blood cells even at concentrations (120 µg mL^−1^) four times higher than MIC_50_ concentration [15]. 

**Figure 1:**
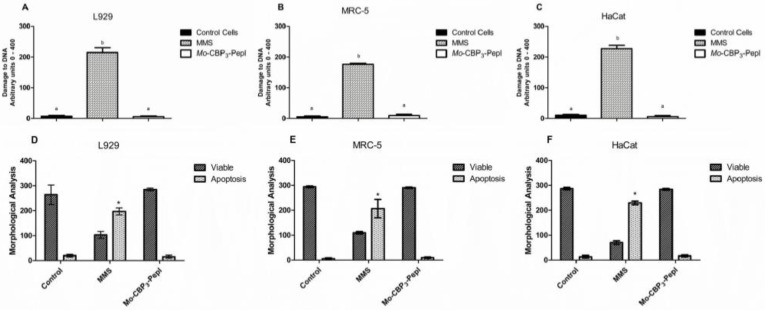
**Assessment of toxicity of *Mo*-CBP3-PepI to human cell lines.** (**A**) L929, (**B**) HaCaT, and (**C**) MRC-5 lines were incubated with synthetic peptide at a concentration of 1 mg mL^−1^ to evaluate the damage to DNA by comet assay. (**D**–**F**) Cell lines were incubated with peptides as described and evaluated for viable cells and cells in apoptosis. MMS (4 × 10^−5^ M) was employed as a positive control for cell toxicity, and healthy cells as a negative control for toxicity. Data are shown as mean ± standard deviation of three independent experiments. * *p* < 0.05. Small letters indicate statistical significance.

In this study, we went further in the analysis of toxicity. *Mo*-CBP_3_-PepI was assayed against other human cells (Figure 1). MTT assay and morphological analysis of human fetal lung fibroblast (MRC-5 line), human keratinocytes (HaCaT line), and L929 fibroblast cells from mice revealed that *Mo*-CBP_3_-PepI did not affect either cell viability and morphology of those cells even at a concentration of 1 mg mL^−1^, which is 32 times higher than MIC_50_ concentration. In contrast, the positive control for damage methyl methanesulfonate (MMS) (4 × 10^–5^ M) led to the death of all cells. It caused severe damage to DNA and nuclei structure indicating the establishment of cell death (Figure 1).

**Figure 2:**
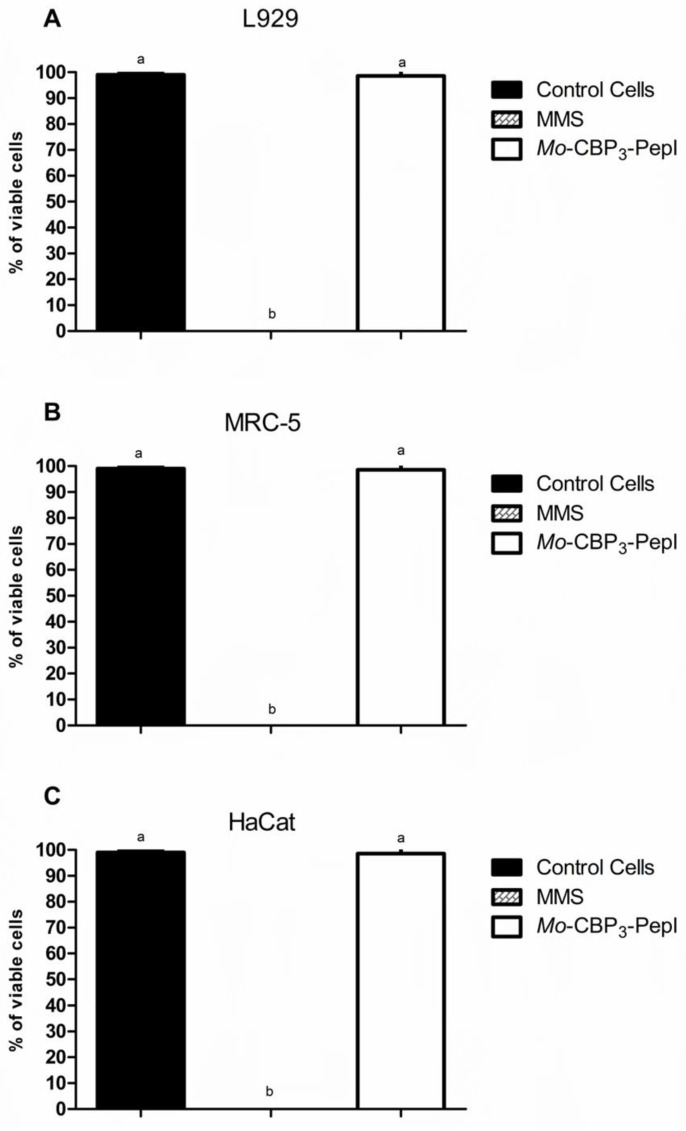
**Evaluation of toxicity of *Mo*-CBP3-PepI to human cell lines by MTT viability assay.** In (**A**) L929, (**B**) HaCaT, and (**C**) MRC-5 lines were incubated with synthetic peptides at a concentration of 1 mg mL^−1^. Methyl methanesulfonate (MMS; 4 × 10^−5^ M) was employed as a positive control for cell toxicity, and healthy cells as a negative control for toxicity. Data are shown as mean ± standard deviation of three independent experiments with significance of *p* < 0.05. Small letters indicate statistical significance.

Another experiment to evaluate damage and fragmentation of DNA caused by *Mo*-CBP_3_-PepI was evaluated by Comet assay (Figure 2) against the same cell lines and at the same concentration. The assay revealed that the DNA of cells treated with peptides presented no damage. In contrast, cells treated with MMS presented with completely damaged DNA (Figure 2). These results assure that the peptide is either safe or presents a low risk for human cells. Other peptides have presented high toxicity to human red blood cells and other human-type cells, such as WRL-68 (liver cells) and NL-20 (lung cells) [23]. 

The synthetic peptides RN7-IN10, RN7-IN9, RN7-IN8, RN7-IN7, and RN7-IN6, derived from indolicidin and ranalexin, presented 50% of hemolytic activity, respectively, at 62.5, 62.5, 125, and 125 µg mL^−1^ [23]. Additionally, the authors revealed that the synthetic peptides RN7-IN10, RN7-IN9, RN7-IN8, RN7-IN7, and RN7-IN6 were toxic to human cell lines such as WRL-68 (liver cells) and NL-20 (lung cells) at a concentration of 125 µg mL^−1^ [23]. These concentrations show that those peptides are more toxic than *Mo*-CBP_3_-PepI. Therefore, based on the results of the toxicity of *Mo*-CBP_3_-PepI, we decided to move forward in the experiment of the mechanism of action. 

### 3.3. Mechanism of Action of Mo-CBP_3_-PepI against K. pneumoniae

#### 3.3.1. Membrane Pore Formation and ROS Overproduction

**Figure 3:**
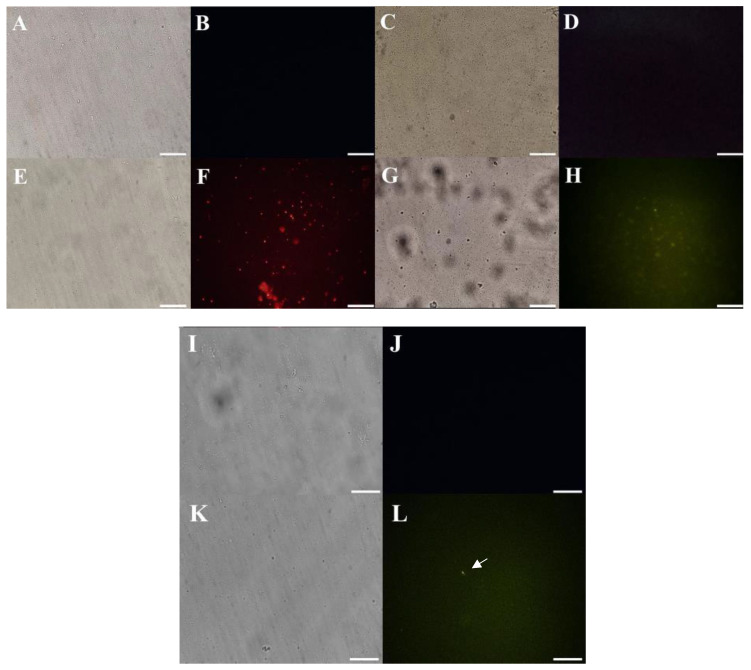
**Mechanism of action of *Mo*-CBP3-PepI assessed by fluorescence microscopy.** In all experiments, *Mo*-CBP3-PepI was used at a concentration of 31.26 μg mL^−1^. (**A**,**B**) control *K. pneumoniae* cells for PI uptake assay. (**E**,**F**) *Mo*-CBP3-PepI-treated *K. pneumoniae* cells for PI uptake assay. Red fluorescence (**F**) indicates pore formation induced by *Mo*-CBP3-PepI. (**C**,**D**) control *K. pneumoniae* cells for FITC-Dextran uptake assay. (**G**,**H**) *Mo*-CBP3-PepI-treated *K. pneumoniae* cells for FITC-Dextran uptake assay. Green fluorescence (**H**) indicates 10-kDa -sized pore formation induced by *Mo*-CBP3-PepI. (**I**,**J**) control *K. pneumoniae* cells for ROS overproduction assay. (**K**,**L**) *Mo*-CBP3-PepI-treated *K. pneumoniae* cells for ROS overproduction assay. Green fluorescence (**L**) indicates pore formation induced by *Mo*-CBP3-PepI. Bars indicate 100 μm.

The mechanisms employed by *Mo*-CBP_3_-PepI against *K. pneumoniae* were evaluated against planktonic lifestyle, given that the biofilm activity was not satisfactory (Table 1). The assay to evaluate the pore in the membrane by PI uptake revealed that the treatment with *Mo*-CBP_3_-PepI induced the pore formation in the membrane of *K. pneumoniae* cells, as revealed by the detected red fluorescence (Figure 3A,B,E,F). The green fluorescence of Dextran-FITC (Figure 3C,D,G,H) indicated that the pore formed by *Mo*-CBP_3_-PepI in the membrane of *K. pneumoniae* cells is at least 10 kDa because the size fluorophore used is 10 kDa.

Membranes are the main target of synthetic or natural peptides [16,24]. This is different from antibiotics, which generally affect a protein leading to the rapid development of resistance. By targeting the membrane of *K. pneumoniae*, *Mo*-CBP_3_-PepI imposes a challenging problem for cells to overcome. Membranes are a complex structure, highly conserved during cell evolution. So, remodeling plasma membrane upon external stress is metabolically expensive and dangerous to cells [14]. 

*Mo*-CBP_3_-PepI, *Mo*-CBP_3_-PepII, and *Mo*-CBP_3_-PepIII are synthetic peptides derived from a chitin-binding protein purified from *Moringa oleifera* [15]. In the same way as Mo-CBP3-PepII and Mo-CBP3-PepIII, Mo-CBP3-PepI can interact with chitin and induce membrane pores in important human pathogenic fungi such as *Candida* spp., *Cryptococcus neoformans,* and *Trichophyton mentagrophytes* [16,17,25]. Here, the mechanisms of action behind the antibacterial activity of *Mo*-CBP3-PepI against *K. pneumoniae* cells were evaluated. The movement of PI (Figure 3A,B,E,F) through the membrane of *K. pneumoniae* revealed by red fluorescence indicates the permeabilization by establishing a pore size of 692.50 Da [26,27]. The size is estimated to be 10 kDa (Figure 3C,D,G,H), given the green fluorescence released by Dextran-FITC.

The questions are, how does *Mo*-CBP_3_-PepI induce pore formation in the membrane of *K. pneumoniae* cells, and why is the pore so big? To answer the first question, it is necessary to revert to the design process of *Mo*-CBP_3_-PepI. During the design process, the *Mo*-CBP_3_-PepI sequence was achieved for reaching three essential features for antimicrobial activity, positive net charge (+1), hydrophobic potential (62%), and probability of 100% to produce a secondary structure in α-helix [13,15,28]. First, the positive net charge of *Mo*-CBP_3_-PepI given by the presence of an arginine residue is essential for the ionic attraction of *Mo*-CBP_3_-PepI to negatively charged peptides in the outlier of *K. pneumoniae* membrane and initial insertion on it [29]. Second, the hydrophobic potential critical for interaction with the hydrophobic core of the membrane’s lipid bilayer is conferred by the presence of apolar amino-acid residues [30]. Third, a secondary structure in α-helix is essential to the attraction and insertion of *Mo*-CBP_3_-PepI into the *K. pneumoniae* [28]. Bioinformatics analysis revealed that *Mo*-CBP_3_-PepI is a cell-penetrating peptide [15]. It is important to notice that *K. pneumoniae* is a Gram-negative bacterium with an outer membrane completely exposed to the *Mo*-CBP_3_-PepI attack by the above mechanism. 

To answer the second question, it is necessary to understand an essential characteristic of antimicrobial peptides, self-association [31]. Self-association is the ability of antimicrobial peptides to interact during the insertion into the membrane, allowing the formation of a huge pore [31]. Based on this result, it is hypothesized the establishment of a barrel-stave model induces pore formation. In this model, a peptide interacts with lipids in the membrane, as described above, to perform the insertion into the membrane. Then, the peptide molecules interact between them to form a huge pore on the membrane [31,32,33]. Lima et al. [16] showed that *Mo*-CBP_3_-PepI induces the formation of a pore of 10 kDa in *C. albicans* cells.

In the same way, the evaluation of ROS overproduction in *K. pneumoniae* cells was evaluated. The treatment of *K. pneumoniae* cells with *Mo*-CBP_3_-PepI led to a slight accumulation of ROS within cells. In contrast, control cells treated with DMSO did not present any ROS production, which was an expected result (Figure 3I,J). ROS are essential to cell development. However, the levels of ROS in the cell have to be regulated because higher accumulation than necessary could be lethal for the cell. The induction of overproduction of ROS could be lethal to cells (Figure 3K,L). The undesired and uncontrolled ROS production and accumulation are associated with damage in important cell life molecules such as proteins, lipids, and DNA, driving cells to death [26]. It is known that *Mo*-CBP_3_-PepI can induce ROS overproduction in *C. albicans* cells [16]. However, the ability to induce ROS in bacterial cells is revealed for the first time. Rowe-Magnus et al. [34] showed that synthetic Cathelicidin-derived peptides induced ROS overproduction in the Gram-negative *Vibrio cholera.* The authors discuss that induction of ROS accumulation was mediated by damage caused in the membrane. Here, we showed that *Mo*-CBP3-PepI could also induce pore formation in the membrane of *K. pneumoniae* and ROS overproduction. So, it is possible to correlate the ROS overproduction induced by *Mo*-CBP3-PepI in *K. pneumoniae* cells with the pore that was formed. 

#### 3.3.2. Scanning Electron Microscopy (SEM)

**Figure 4:**
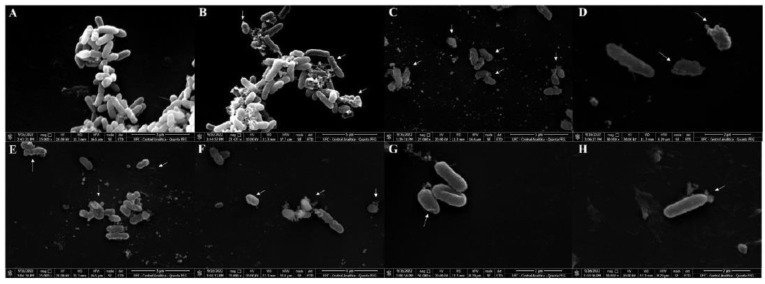
**Scanning electron microscope of *K. pneumoniae* cells treated with *Mo*-CBP3-PepI.** (**A**) control *K. pneumonia* cells treated with DMSO-NaCl solution. (**B**–**H**) *K. pneumoniae* cells treated with *Mo*-CBP3-PepI 31.26 μg mL^−1^.

SEM analysis was employed to provide more insights into the effect of *Mo*-CBP_3_-PepI on *K. pneumoniae* morphology (Figure 4). As expected, control *K. pneumoniae* cells treated with DMSO presented a healthy morphology with no damage on the surface (Figure 4A). However, *Mo*-CBP_3_-PepI-treated *K. pneumoniae* cells presented several lethal damages on the surface (Figure 4B–H). After treatment with *Mo*-CBP_3_-PepI, *K. pneumoniae* cells presented cells completely broken with damage to the cell wall, such as depressions, abnormal morphology, roughness, and an irregular cell surface (Figure 4B,C—white arrows), and in many cases, it is possible to see that the extravasation of cytoplasmic content may occur due to the pores formed in the membrane (Figure 4D–F,H—white arrows). Interestingly, *Mo*-CBP_3_-PepI-treated *K. pneumoniae* cells presented structure-like depressions, broken cell walls, and contorted cells (Figure 4G). 

SEM analysis corroborates the damage suggested by fluorescence microscopy. As revealed by fluorescence microscopy, *K. pneumoniae* cells present pores in the membrane after treatment with *Mo*-CBP_3_-PepI. SEM analysis revealed several areas of damage to the structure of *K. pneumoniae,* including the loss of internal content mediated by pores. Mo-CBP3-PepI induced the same damage in *Candida* cells [16]. For example, *Mo*-CBP_3_-PepIII, a closely related peptide of *Mo*-CBP_3_-PepI, also damaged the morphology of *Staphylococcus aureus* [15].

### 3.4. Proteomic Profile of K. pneumoniae Cells Treated with Mo-CBP_3_-PepI

#### 3.4.1. Overview

**Figure 5:**
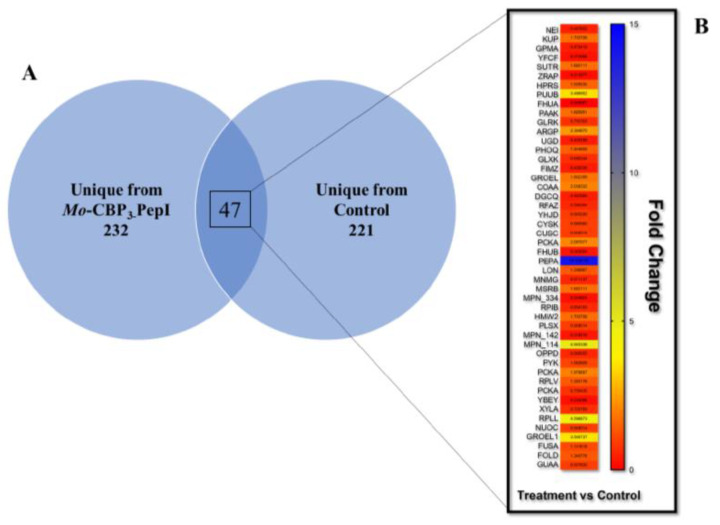
**Distribution of *K. pneumoniae* proteins obtained after treatment with *Mo*-CBP3-PepI.** (**A**) Venn diagram shows the dispersion of total proteins of control and *Mo*-CBP3-PepI-treated cells. (**B**) Represents the fold-change value of overlapping proteins, which were found in both groups. The vertical bar indicates the color scale according to each protein’s fold-change value.

Proteomic analysis is a powerful technique employed to provide an overview of what happens in cells after treatment with peptides [35,36,37,38,39]. The proteomic response to peptides has been analyzed in *Escherichia coli* K12 [40], *Bacillus subtilis* [35], and *Clostridioides difficile* [38]. In many cases, proteomic analysis has been performed to understand the behavior of the multidrug-resistant bacteria’s response to antibiotics [36]. Here, proteomic analysis was employed to overview protein changes in *K. pneumoniae* after treatment with *Mo*-CBP_3_-PepI. In total, 547 were successfully identified (Figure 5). In total, 279 proteins were identified in *Mo*-CBP_3_-PepI-treated *K. pneumoniae* cells and 268 in control *K. pneumoniae* cells (Figure 3A; Tables S1 and S2). Among exclusive proteins, 232 unique proteins were from *Mo*-CBP_3_-PepI-treated *K. pneumoniae* cells and 221 were unique from control *K. pneumoniae* cells (Figure 5A; Tables S1 and S2). 

Besides the unique proteins, which are those exclusively found in one group, there were proteins detected in both groups, and they were shared proteins. To understand the patterns of differential accumulation of these proteins, a fold-change rule was applied, taking into account the intensity of protein *Mo*-CBP_3_-PepI/Control *K pneumoniae* cells. 

Among the overlapping proteins, which were found in both groups, proteins with a fold-change value ≥ 1.5 (*p* < 0.05, Tukey’s test) [41] were considered up-accumulated (increased the abundance), and proteins with a fold-change value ≤ 0.5 (*p* < 0.05, Tukey’s test) were considered down-accumulated (decreased the abundance). For example, endonuclease 8 has a fold-change value of 0.287 and considered with a reduced abundance in cells treated with *Mo*-CBP_3_-PepI compared to control cells. 

Forty-seven proteins common to both groups, 19 up-accumulated, 19 down-accumulated, and 9 did not change when comparing *Mo*-CBP_3_-PepI-treated with control cells (Figure 5B). 

**Figure 6:**
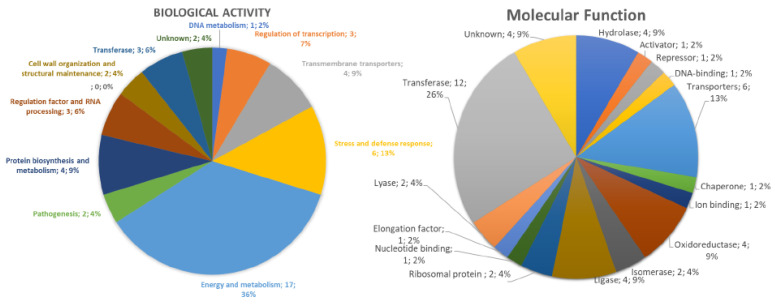
**Classification proteins from *K. pneumoniae* cells identified by LC-ESI-MS/MS analysis.** The overlapping proteins (found in both control and treated) were classified based on biological process and molecular function.

The Gene ontology classification of proteins shared by *Mo*-CBP3-PepI- and control- *K. pneumoniae* cells revealed 11 and 16 groups of proteins, respectively, for biological activity and molecular function (Figure 6). Regarding the biological activity, the group that held the highest number of identified proteins was Energy and Metabolism with 36% of total proteins and the DNA metabolism group held the lowest number with 2% of the total identified proteins (Figure 6). In the case of molecular function, the transferase group possessed the highest amount, 26%, of identified proteins. Many groups, such as chaperone and ion binding, have a small number of proteins, 2% of the total identified included (Figure 6). 

The protein groups involved in regulating transcription, transmembrane transporters, stress, and Defense Response, Energy and Metabolism, Pathogenesis, Protein Biosynthesis, Metabolism, Cell wall organization, and structural maintenance and transferase were composed of proteins that are both up- and down-accumulated (Table 2). In contrast, the groups Regulation Factor and RNA Processing and DNA metabolism are composed of proteins that decrease the abundance in *K. pneumoniae* cells after treatment with *Mo*-CBP_3_-PepI (Table 2 and Figure 5B Fold-Change). On the other hand, the downregulated proteins were related to regulation factors and transferase (Figure 5B—Fold-Change). 

#### 3.4.2. DNA Metabolism-Related Proteins

In this group, one protein was found in both *Mo*-CBP_3_-PepI and DMSO groups, Endonuclease 8 (Table 2), which was down-accumulated in *K. pneumoniae* cells treated with *Mo*-CBP_3_-PepI compared to DMSO cells (control). Endonuclease 8 is an enzyme involved in the DNA repair process after damage by oxidation by ROS [42,43,44]. The reduction in the abundance of Endonuclease 8 in cells treated with *Mo*-CBP_3_-PepI is attractive because it noticed a higher accumulation of ROS (Figure 3) in those cells. So, the high accumulation of ROS and reduction in accumulation of the Endonuclease 8 suggest that DNA from *K. pneumoniae* cells is being damaged by ROS induced by *Mo*-CBP_3_-PepI [26]. 

The proteins UvrA, UvrB, and UvrC, were exclusive from *K. pneumoniae* cells treated with *Mo*-CBP_3_-PepI (Table S1). Based on that, it is feasible to suggest that *Mo*-CBP_3_-PepI induced damage in the DNA of *K. pneumoniae* because these enzymes repair both strands of DNA [45]. Interestingly, only in the control cells (Table S2), but not in treated cells, the protein DNA repair protein RecN was detected as the cell’s first line to protect the DNA from damage [46]. Somehow, *Mo*-CBP_3_-PepI induces a down-accumulation in *K. pneumoniae* cells that, combined with ROS accumulation, leads to DNA damage and cell death.

#### 3.4.3. Stress and Defense Response Related Proteins

In this group, one protein deserved attention, Peptide methionine sulfoxide reductase MsrB was highly accumulated in *K. pneumoniae* cells treated with *Mo*-CBP_3_-PepI compared to control cells with a fold-change value of 14.104 (Table 2). The MsrB protein is a highly conserved protein essential in the cell defense mechanism against high ROS levels, and it works in the repair of inactivated protein by ROS [47]. The increase in MsrB in cells treated with *Mo*-CBP_3_-PepI compared to control agrees with the high accumulation of ROS in cells (Figure 3). Proteins and other vital molecules in the cell are attacked and inactivated by ROS [26]. The increased abundance of MsrB protein indicates severe damage in proteins of *K. pneumoniae* cells after treatment with *Mo*-CBP_3_-PepI and that cells are trying to recover from this stress. The MsrB proteins are involved in recovering proteins oxidized by ROS species [26].

Proteomic analysis of cells revealed a reduction in the accumulation of an important protein, Lon protease, in cells treated with *Mo*-CBP_3_-PepI compared to control cells (Table 2). Lon protease is a multifunctional, highly conserved ATP-dependent serine protease involved in protein turnover in bacterial cells [48,49]. Lon protease degrades either natural or ROS-induced misfolded proteins leading to free amino acids to produce new functional proteins [48,50]. The reduction in the abundance of Lon protease in *Mo*-CBP_3_-PepI-treated cells may suggest an accumulation of misfolded proteins that mitigate the chance of responding to the stress imposed by *Mo*-CBP3-PepI. Additionally, the Lon protease is essential to encapsulation, motility, heat-shock response, persister formation and drug resistance, and virulence factor production [51,52,53,54,55,56]. By inducing the reduction in accumulation of Lon protease in *K. pneumoniae* cells, *Mo-CBP3-PepI* dramatically reduced the chances of the cell to respond to stress and inhibit several essential processes to cell normal function leading to death.

The proteins unique from control cells were identified, such as multidrug resistance protein MdtN, UPF0194 membrane protein YbhG, and multidrug resistance protein EmrK (Table S2). MdtN is a protein involved in the resistance against puromycin and acriflavine [55], EmrK is a part of the efflux pump involved in multidrug resistance [57], and YbhG is involved in resistance to chloramphenicol [58]. This is an exciting result because the absence of these in the *Mo-CBP3-PepI*-treated cells indicates that they became susceptible to these antibiotics again, which is an important outcome. 

#### 3.4.4. Protein Biosynthesis and Metabolism Related Proteins

The analysis of proteins related to protein biosynthesis and metabolism revealed a quite complex scenario in *K. pneumoniae* cells after treatment with *Mo*-CBP_3_-PepI (Table 2; Tables S1 and S2). For example, among the overlapping proteins, the 50S ribosomal protein L22 and 50S ribosomal protein L7/L12, respectively, showed a reduction and increased abundance in *K. pneumoniae* cells treated with *Mo*-CBP_3_-PepI compared to control cells (Table 2). The 50S ribosomal protein L22 is a vital core protein of bacterial ribosomes involved in the aggregation and stabilization of ribosomal proteins to form the ribosome in bacteria [59,60]. The L22 subunit is so important to bacterial ribosomes that it is the target of antibiotics such as macrolides [59,60]. The reduction in abundance in this protein induced by *Mo-CBP3-PepI* indicates a destabilization of bacterial ribosomes leading to the inhibition of protein synthesis in bacteria.

An increase in the abundance of 50S ribosomal protein L7/L12 in cells treated with *Mo-CBP3-PepI* (Table 2) was seen, compared to the control. The increase in this protein is perhaps a mechanism of the cell to supply the deficiency of the ribosomal protein L22. However, the L7/L12 is a GTPase protein involved in the process such as translation initiation, elongation, and termination by the mature 70S ribosome [61]. However, increasing this protein will not help cells perform protein synthesis without the L22 subunit.

An interesting result came out by evaluating the unique proteins, *Mo*-CBP_3_-PepI and control cells (Table 1 and Table 2). Among the proteins exclusively detected in *K. pneumoniae* cells treated with *Mo*-CBP_3_-PepI are Cysteine-tRNA ligase, Leucine-tRNA ligase, Serine-tRNA ligase, Valine-tRNA ligase, Glutamine-tRNA, Phenylalanine-tRNA ligase alpha subunit, Valine-tRNA ligase, Proline-tRNA ligase, Alanine-tRNA ligase, and Threonine-tRNA ligase (Table S1). All these proteins are involved in amino acid delivery to ribosomes during protein synthesis. The increase in these proteins’ abundance indicates a cell attempt to either increase or maintain the protein synthesis at normal levels to allow cells to fight back against insults imposed by *Mo*-CBP3-PepI. 

Proteins are important to all living organisms, and bacteria must understand what is happening in their environment to respond accordingly. Proteins make all this happen. To respond to stress agents, such as *Mo*-CBP3-PepI, *K. pneumoniae* must reprogram all its protein profiles to produce defense proteins [62]. For example, *K. pneumoniae* cells should produce scavenger proteins to defend themselves from ROS overproduction, but that is impossible. That happens because *Mo*-CBP3-PepI reduced the abundance of an important protein for ribosomal activity. As a consequence, *K. pneumoniae* cells could not produce scavenger proteins, thus leading to ROS accumulation and damage to DNA (as reported above) and damage to other proteins leading the cell to death as revealed by the damage present in fluorescence and scanning electron microscopy. 

#### 3.4.5. Regulation Factor and RNA Processing Related Proteins

In this group of proteins, the protein elongation factor (Q7NAV3) G that decreases in abundance in *K. pneumoniae* treated with *Mo*-CBP3-PepI deserves attention (Table 2). By looking into unique proteins from control cells (Table S2), one isoform of elongation factor G (Q492B1) and other factors, such as Elongation factor Tu 1 and Elongation factor 4, are present but disappear completely in treated versus control cells. Elongation factor G is important for the translocation process during prokaryotic protein synthesis, and it uses the energy held in GTP to interact with tRNA and mRNA [63]. The decrease in the abundance of Elongation factor G led to the shutdown of protein synthesis in *K. pneumoniae* cells. As discussed above in the protein metabolism section, the protein synthesis in *K. pneumoniae* cells is dramatically affected by Mo-CBP3-PepI, giving them no chance to respond to the stress imposed by the peptide.

#### 3.4.6. Cell Wall Organization and Structure Maintenance Related Proteins

In this group of proteins, important proteins were exclusively found in *K. pneumoniae* cells treated with *Mo*-CBP_3_-PepI (Table S1), which are soluble lytic murein transglycosylase, D-alanyl-D-alanine carboxypeptidase DacB, Cell shape-determining protein MreB, Probable L, D-transpeptidase ErfK/SrfK, Cell shape-determining protein MreC, Murein tetrapeptide carboxypeptidase, Murein DD-endopeptidase MepH, Sensor protein LytS, D-alanine–D-alanine ligase, and Inner membrane protein YdcZ. All are involved in cell wall turnover, cell structure maintenance, and shape stabilization [64,65,66]. The increased abundance of these proteins indicates that K. pneumoniae cells are suffering from stress in the cell wall imposed by *Mo*-CBP3-PepI and are trying to overcome the stress. However, as revealed by SEM analysis (Figure 4), *K. pneumoniae* cells treated with *Mo*-CBP_3_-PepI presented damage to the cell wall and cell morphology. It has been related that *Mo*-CBP3-PepI can bind to the cell wall of the yeast *C. albicans* [16].

The bacterial cell wall is a crucial component of the cell involved in mechanical defense against several environmental stresses [67]. During stress, the cell must recover the cell wall every time that damage occurs. The exclusive detection of this protein in cells treated with *Mo*-CBP_3_-PepI indicates that it is imposing stress on the cell wall and is trying to recover, but as revealed by microscopic analysis (Figure 3 and Figure 4).

#### 3.4.7. Transferase-Related Proteins

In this group, one protein is relevant, the sensor histidine kinase HprS, which presented a reduced abundance in cells treated with *Mo*-CBP3-PepI compared to the control cells (Table 2). Histidine kinase sensors are environmental elements employed by bacteria to sense the environment and respond accordingly [68,69]. These sensors are mainly responsible for perceiving and responding to oxidative stress [70]. Here, the reduction in the abundance of HprS agreed with the high accumulation of ROS (Figure 3) in *K. pneumoniae* cells treated with *Mo*-CBP3-PepI. This result shows that *Mo*-CBP3-PepI imposes two stresses on *K. pneumoniae* cells. First is the induction of accumulation of ROS at higher levels; second is the reduction in the protein accumulation involved in the perception and response of stress caused by ROS. In this case, *Mo*-CBP3-PepI simultaneously induces stress and inhibits the cell’s ability to develop a response to it. 

#### 3.4.8. Cell Redox Homeostasis-Related Proteins

This group is particularly important given the scenario of high levels of ROS accumulation in *K. pneumoniae* cells induced by *Mo*-CBP3-PepI. In this group, where no overlapping proteins found, only exclusive proteins either from *Mo*-CBP3-PepI-treated or control *K. pneumoniae* cells (Tables S1 and S2). For example, Alkyl hydroperoxide reductase C and Alkyl hydroperoxide reductase subunit F were only detected in control *K. pneumoniae* (Table S2). Thiol-peroxidases are responsible for the scavenging of H_2_O_2_, protecting the bacterial cell from toxic levels of endogenously H_2_O_2_ [71,72]_._

Interestingly, these enzymes were not detected in cells treated with *Mo*-CBP_3_-PepI, indicating a complete depletion of these enzymes. The absence of these enzymes in treated cells might lead to the accumulation of H_2_O_2_ (Figure 3), as revealed by fluorescence microscopy. To cope with the high levels of ROS induced by peptides, *K. pneumoniae* cells increase the abundance of a catalase-peroxidase enzyme, only detected in treated cells, which is involved in the defense mechanism against high levels of ROS [71]. Our data revealed that even though *K. pneumoniae* cells treated with *Mo*-CBP_3_-PepI increased the abundance of a catalase enzyme to remove the excess ROS, those scavenger enzymes are not enough to prevent the damage produced by high levels of H_2_O_2_ once microscopic analysis shows damage to the cell structure and many enzymes suggest damage in DNA and proteins.

**Figure 7:**
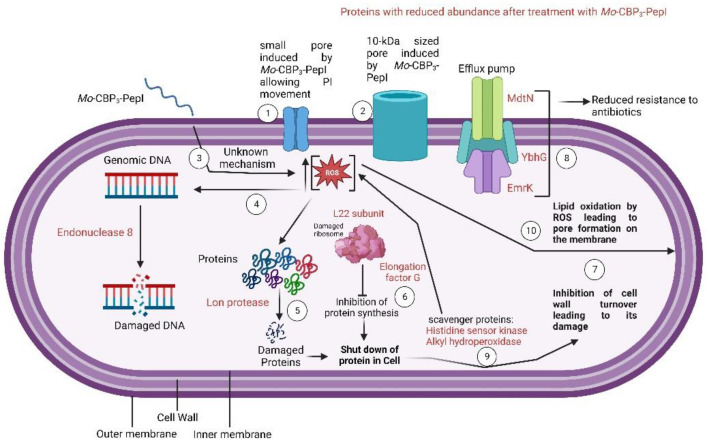
Scheme showing the mechanisms of antibacterial action of *Mo*-CBP3-PepI against *K. pneumoniae*. This scheme was produced only with proteins that decreased in abundance. The numbers explain important process. All them are explained in the text.

The mechanisms by which *Mo*-CBP_3_-PepI induced damage and death in *K. pneumoniae* cells are many and quite complex. Based on that, a scheme (Figure 7) was produced to provide an overview of all processes induced by *Mo*-CBP_3_-PepI that lead K. pneumoniae to death. (1) As revealed by PI uptake, *Mo*-CBP_3_-PepI can induce the formation of tiny pores on the membrane of *K. pneumoniae*. Additionally, FITC-Dextran revealed the presence of a 10-kDa sized pore (2); (3) the interaction of *Mo*-CBP_3_-PepI led to the accumulation of high levels of ROS inside the cell; (4) ROS accumulation led to damage to DNA; (5) The high levels of ROS led to damage to proteins leading to misfolding and degradation; (6) As revealed by proteomic analysis, *Mo*-CBP_3_-PepI induced a reduction in the abundance of proteins related to proteins and, together with the events in (5), led to a shutdown in protein in *K. pneumoniae* cells; (7) The shutdown in protein inhibits the cell wall turnover leading to damage, as revealed by SEM analysis (Figure 4); (8) the shutdown in protein levels is also responsible for reducing proteins related to antibiotic resistance; (9) There is also a reduction in a group of proteins involved in early response to ROS accumulation leading to the accumulation of ROS at higher levels; (10) The higher levels of ROS led to damage to DNA (3), proteins (5), and lipids on the membrane (10). 

## 4. Conclusions

Altogether the data presented here indicate an intricated and coordinated sequence of events induced by *Mo*-CBP_3_-PepI that could drive *K. pneumoniae* cells to death. All these complex mechanisms of action are also difficult for *K. pneumoniae* to develop resistance because they present multiple targets simultaneously. Based on that, it is feasible to suggest that our peptide is a potential candidate for developing new strategies to cope with *K. pneumoniae* resistance. 

## Figures and Tables

**Table 1:** Cell viability and antibiofilm potential of *Mo*-CBP3-PepI against *K. pneumoniae*.

^a^ MIC_50_ of *Mo*-CBP_3_-PepI toward *K. pneumoniae*			
Treatments		Antibiofilm Potential		
	Cell Viability (%)	Inhibition of Biofilm Formation (%)	Degradation of Preformed Biofilm (%)
DMSO	100 ± 0.005	0	0
*Mo*-CBP_3_-PepI	47.54 ± 0.008	11.87 ± 0.001	0

^a^ The MIC50 concentration was 31.25 μg mL^−1^, as defined by Oliveira et al. [1]. Cell viability assay was executed with planktonic cells.

**Table 2:** Differentially accumulated proteins identified by ESI-LC-MS/MS.

Protein Name	ID (Uniprot)	Organism Reference	Cellular Compartment	Fold Change *Mo*-CBP_3_-PepI vs. DMSO
**DNA metabolism**				
Endonuclease 8	P50465	*Escherichia coli* (strain K12)	Cytoplasm	0.287
**Regulation of transcription**				
HTH-type transcriptional regulator SutR	P77626	*Escherichia coli* (strain K12)	Cytoplasm	2.566
HTH-type transcriptional regulator ArgP	P0A8S1	*Escherichia coli* (strain K12)	Cytoplasm	2.364
Fimbriae Z protein	P0AEL8	*Escherichia coli* (strain K12)	Cytoplasm	0.690
**Transmembrane transporters**				
Low affinity potassium transport system protein kup	P63183	*Escherichia coli* (strain K12)	Plasma membrane	1.704
Ferrichrome outer membrane transporter/phage receptor	P06971	*Escherichia coli* (strain K12)	Plasma membrane	1.063
Cation efflux system protein CusC	P77211	*Escherichia coli* (strain K12)	cell outer membrane	0.920
Iron (3+)-hydroxamate import system permease protein FhuB	P06972	*Escherichia coli* (strain K12)	Plasma membrane	0.948
**Stress and Defense Response**				
Zinc resistance-associated protein	P0AAA9	*Escherichia coli* (strain K12)	Periplasm space	1.682
Chaperonin GroEL	P0A6F5	*Escherichia coli* (strain K12)	Cytoplasm	1.444
Undecaprenyl-diphosphatase	Q2KX31	*Bordetella avium (strain 197N)*	Plasma membrane	1.340
Peptide methionine sulfoxide reductase MsrB	P75129	*Mycoplasma pneumoniae* (strain ATCC 29342/M129)	Cytoplasm	14.104
Periplasmic trehalase	Q4UZ12	*Xanthomonas campestris pv. campestris* (strain 8004)	Periplasmatic space	0.937
Lon protease	P78025	*Mycoplasma pneumoniae* (strain ATCC 29342/M129)	Cytoplasm	0.342
**Energy and Metabolism**				
2.3-bisphosphoglycerate-dependent phosphoglycerate mutase	P62707	*Escherichia coli* (strain K12)	Cytoplasm	0.519
Gamma-glutamylputrescine oxidoreductase	P37906	*Escherichia coli* (strain K12)	Cytoplasm	0.985
2-succinyl-5-enolpyruvyl-6-hydroxy-3-cyclohexene-1-carboxylate synthase	P17109	*Escherichia coli* (strain K12)	Plasma membrane	3.486
UDP-glucose 6-dehydrogenase	P76373	*Escherichia coli* (strain K12)	Cytoplasm	0.902
Glycerate 3-kinase	P77364	*Escherichia coli* (strain K12)	Cytoplasm	0.745
Phenylacetate-coenzyme A ligase	P76085	*Escherichia coli* (strain K12)	Cytoplasm	1.666
Pantothenate kinase	P0A6I3	*Escherichia coli* (strain K12)	Cytoplasm	0.548
Pyruvate kinase	P78031	*Mycoplasma pneumoniae* (strain ATCC 29342/M129)	Membrane	3.139
Phosphoenolpyruvate carboxykinase (ATP)	A8AQV7	*Escherichia coli* (strain K12)	Cytoplasm	0.990
Xylose isomerase	B5ZQV6	*Rhizobium leguminosarum bv. trifolii* (strain WSM2304)	Cytoplasm	1.062
Bifunctional protein FolD	Q88WM8	*Lactiplantibacillus plantarum* (strain ATCC BAA-793/NCIMB 8826/WCFS1)	Periplasmic space	0.726
GMP synthase (glutamine-hydrolyzing)	Q6APU2	*Desulfotalea psychrophila* (strain LSv54/DSM 12343)	Cytoplasm	4.596
Sensor histidine kinase GlrK	P52101	*Escherichia coli* (strain K12)	Cell inner membrane	0.752
Sensor protein PhoQ	P23837	*Escherichia coli* (strain K12)	Plasma membrane	0.823
Putative ABC transporter ATP-binding protein MPN_334	P75444	*Mycoplasma pneumoniae* (strain ATCC 29342/M129)	Plasma membrane	1.336
Oligopeptide transport ATP-binding protein OppD	P75552	*Mycoplasma pneumoniae* (strain ATCC 29342/M129)	Plasma membrane	0.948
Pantothenate synthetase	B8I2Z3	*Ruminiclostridium cellulolyticum* *(strain ATCC 35319/DSM 5812/JCM 6584/H10)*	Cytoplasm	1.142
**Pathogenesis**				
Lipopolysaccharide core biosynthesis protein RfaZ	P27241	*Escherichia coli* (strain K12)	Cytoplasm	2.038
Inner membrane protein YhjD	P37642	*Escherichia coli* (strain K12)	Plasma membrane	0.362
**Protein Biosynthesis and Metabolism**				
50S ribosomal protein L22	A5IYY1	*Mycoplasmopsis agalactiae*	Large ribosomal subunit	0.187
50S ribosomal protein L7/L12	P0A466	*Aquifex aeolicus* (strain VF5)	Large ribosomal subunit	1.979
Diaminopimelate decarboxylase	Q8K9C4	*Buchnera aphidicola subsp. Schizaphis graminum* (strain Sg)	Cytoplasm	3.546
Cysteine synthase A	P0ABK5	*Escherichia coli* (strain K12)	Cytoplasm	0.596
**Regulation Factor and RNA Processing**				
tRNA uridine 5-carboxymethylaminomethyl modification enzyme MnmG	P75221	*Mycoplasma pneumoniae* (strain ATCC 29342/M129)	Cytoplasm	0.487
Elongation factor G	Q7NAV3	*Mycoplasma gallisepticum (strain R(low/passage 15/clone 2))*	Cytoplasm	0.234
Methylenetetrahydrofolate--tRNA-(uracil-5-)-methyltransferase TrmFO	A4WRQ2	*Cereibacter sphaeroides* (strain ATCC 17025/ATH 2.4.3)	Cytoplasm	0.948
**Cell wall organization and structural maintenance**				
Cytadherence high molecular weight protein 2	P75471	*Mycoplasma pneumoniae* (strain ATCC 29342/M129)	Cytoplasm	1.682
Mgp-operon protein 3	Q50341	*Mycoplasma pneumoniae* (strain ATCC 29342/M129)	Plasma membrane	0.654
**Transferase**				
Glutathione S-transferase YfcF	P77544	*Escherichia coli* (strain K12)	Cytoplasm	0.273
Phosphate acyltransferase	P75232	*Mycoplasma pneumoniae* (strain ATCC 29342/M129)	Cytoplasm	3.096
Sensor histidine kinase HprS	P76339	*Escherichia coli* (strain K12)	Plasma membrane	0.213
**Unknown**				
Probable cytosol aminopeptidase	P75206	*Mycoplasma pneumoniae* (strain ATCC 29342/M129)	Cytoplasm	2.097
Putative acetyltransferase MPN_114	P75448	*Mycoplasma pneumoniae* (strain ATCC 29342/M129)	Unknown	1.703

## Data Availability

The data supporting this study’s findings are available on request from the corresponding author.

## Supplementary Materials

The following supporting information can be downloaded at: https://www.mdpi.com/article/10.3390/antibiotics11121753/s1, Figure S1: Purity and quality of synthesis process of Mo-CBP3-PepI. (A) reverse-phase high performance liquid chromatography to the purity of Mo-CBP3-PepI. (B) MS/MS data analysis corroborating the result of HPLC analysis; Table S1: Mo-CBP3-PepI group unique proteins identified by ESI-LC-MS/MS.
