# Supplementary material for: New Insights into the Mechanism of Antibacterial Action of Synthetic Peptide Mo-CBP3-PepI against Klebsiella pneumoniae
Source: Antibiotics (Basel). 2022 Dec 4;11(12):1753. doi: 10.3390/antibiotics11121753 (PMC9774128; doi:10.3390/antibiotics11121753)
Supplement: Supplementary file 1 [file antibiotics-11-01753-s001.zip › antibiotics-2065623-supplementary.pdf]

A

B

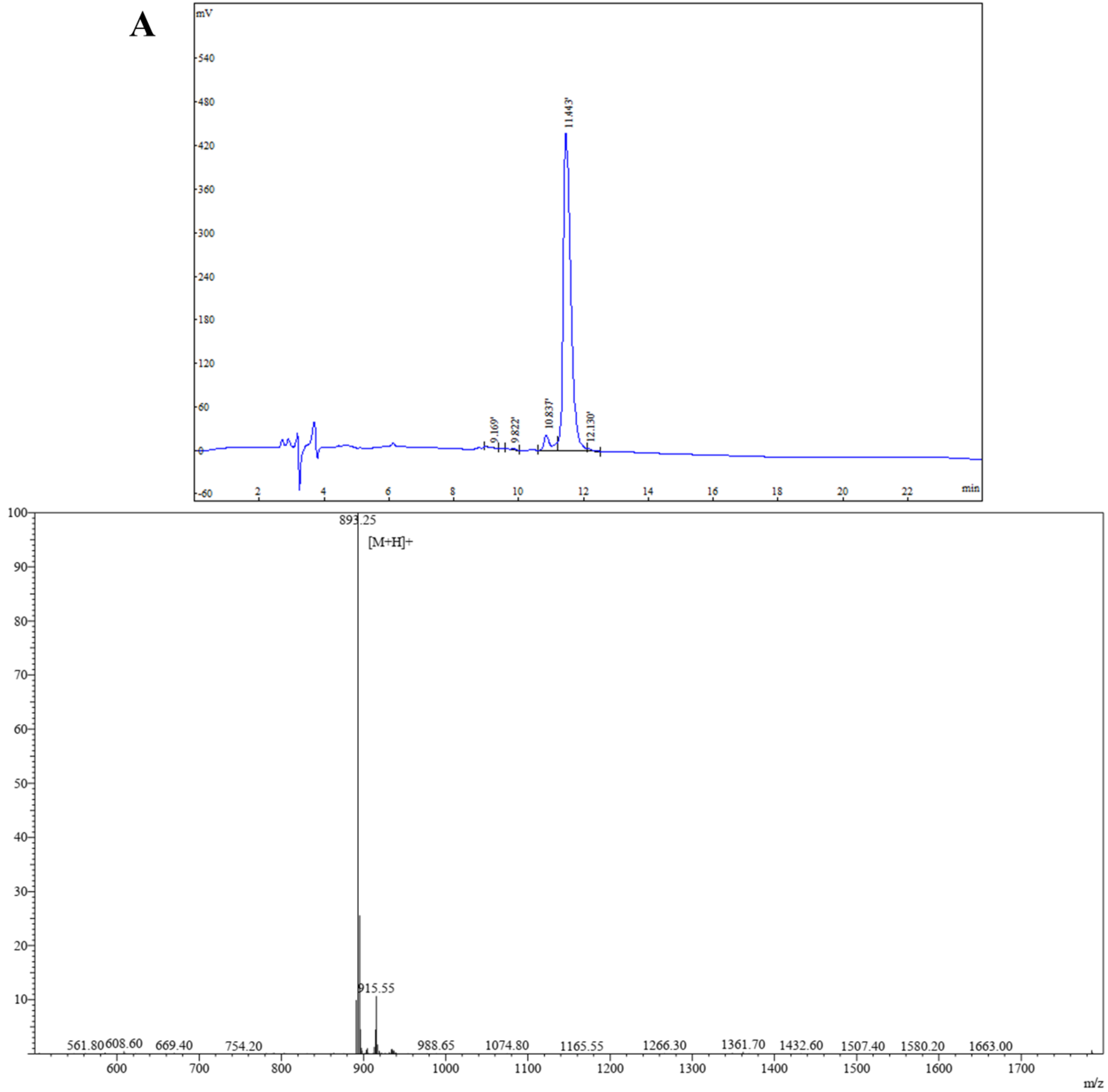

mV
540
480
420
360
300
240
180
120
60
0
-60
2
4
6
8
10
12
14
16
18
20
22
min
9.169'
9.822'
10.837'
11.443'
12.130'
893.25
[M+H]+
915.55
561.80
608.60
669.40
754.20
988.65
1074.80
1165.55
1266.30
1361.70
1432.60
1507.40
1580.20
1663.00
100
90
80
70
60
50
40
30
20
10
600
700
800
900
1000
1100
1200
1300
1400
1500
1600
1700
m/z

Supplementary Figure S1. Purity and quality of synthesis process of *Mo*-CBP$_3$-PepI. (A) reverse-phase high performance liquid chromatography to the purity of *Mo*-CBP$_3$-PepI. **(B)** MS/MS data analysis corroborating the result of HPLC analysis.

Supplementary Table S1 – *Mo*-CBP$_3$-PepI group unique proteins identified by ESI-LC-MS/MS

Protein Name	ID Uniprot	Organism Reference	Cellular Compartment
**<u>Carbohydrate metabolism</u>**			
3-dehydro-D-guloside 4-epimerase	P76044	*Escherichia coli* (strain K12)	Cytoplasm
D-xylonate dehydratase YjhG	P39358	*Escherichia coli* (strain K12)	Cytoplasm
Apulose kinase	Q6D5T8	*Pectobacterium atrosepticum* (strain SCRI 1043 / ATCC BAA-672)	Cytoplasm
<u>Lipid metabolism</u>			
7alpha-hydroxysteroid dehydrogenase	P0AET8	*Escherichia coli* (strain K12)	Cytoplasm
Acetyl-coenzyme A carboxylase carboxyl transferase subunit beta	P0A9Q5	*Escherichia coli* (strain K12)	Cytoplasm
Fatty acid oxidation complex subunit alpha	P21177	*Escherichia coli* (strain K12)	Cytoplasm
3-ketoacyl-CoA thiolase	Q48GW4	*Pseudomonas savastanoi pv. phaseolicola* (strain 1448A / Race 6)	Cytoplasm
Lipid-A-disaccharide synthase	B7V7U5	*Pseudomonas aeruginosa* (strain LESB58)	Cytoplasm
Glycerol-3-phosphate acyltransferase	Q1GYU3	*Methylobacillus flagellatus* (strain KT / ATCC 51484 / DSM 6875)	Plasma membrane
3-hydroxyacyl-[acyl-carrier-protein] dehydratase FabZ	B9DY11	*Clostridium kluyveri* (strain NBRC 12016)	Cytoplasm
4-hydroxy-2-oxovalerate aldolase 1	Q47G67	*Dechloromonas aromatica* (strain RCB)	Cytoplasm

Cell Redox Homeostasis			
Hydroxylamine reductase	P75825	*Escherichia coli* (strain K12)	Cytoplasm
Periplasmic serine endoprotease DegP	P0C0V0	*Escherichia coli* (strain K12)	Cytoplasm
Protein-methionine-sulfoxide reductase catalytic subunit MsrP	P76342	*Escherichia coli* (strain K12)	Periplasmic space
Catalase-peroxidase	Q1GCU3	*Ruegeria sp.* (strain TM1040)	Cytoplasm
Stress and Defense Response			
Probable L,D-transpeptidase YcbB	P22525	*Escherichia coli (strain K12)*	Plasma membrane
Zinc resistance-associated protein	P0AAA9	*Escherichia coli* (strain K12)	Periplasmic space
Chaperone protein HtpG	P0A6Z3	*Escherichia coli* (strain K12)	Cytoplasm
Co-chaperonin GroES	B5F2K9	*Salmonella agona (strain SL483)*	Cytoplasm
ATP-dependent Clp protease ATP-binding subunit ClpX	Q2RU44	*Rhodospirillum rubrum* (strain ATCC 11170 / ATH 1.1.1 / DSM 467 / LMG 4362 / NCIMB 8255 / S1)	Cytoplasm
Chaperone protein DnaJ	Q5FGQ8	*Ehrlichia ruminantium* (strain Gardel)	Cytoplasm
Energy and Metabolism			
Multiphosphoryl transfer protein 1	P77439	*Escherichia coli* (strain K12)	Cytoplasm
Alpha-D-ribose 1-methylphosphonate 5-triphosphate synthase subunit PhnG	P16685	*Escherichia coli* (strain K12)	Unknown
1-deoxy-D-xylulose 5-phosphate reductoisomerase	P45568	*Escherichia coli* (strain K12)	Cytoplasm
Aspartate ammonia-lyase	Q8XDS0	*Escherichia coli* O157:H7	Cytoplasm

Trehalose synthase	Q1ARU5	*Rubrobacter xylanophilus* (strain DSM 9941 / NBRC 16129 / PRD-1)	Cytoplasm
Cytochrome bc1 complex Rieske iron-sulfur subunit	Q9X807	*Streptomyces coelicolor* (strain ATCC BAA-471 / A3(2) / M145)	Plasma membrane
Fructose-1,6-bisphosphatase class 1 2	B2JVN8	*Paraburkholderia phymatum* (strain DSM 17167 / CIP 108236 / LMG 21445 / STM815)	Cytoplasm
1,4-alpha-glucan branching enzyme GlgB	A7FNX5	*Yersinia pseudotuberculosis serotype O:1b* (strain IP 31758)	Cytoplasm
ATP synthase subunit a	Q4FPE9	*Pelagibacter ubique* (strain HTCC1062)	Plasma membrane
Glycogen synthase	B1I4F5	*Desulforudis audaxviator* (strain MP104C)	Cytoplasm
Lipoamide acyltransferase component of branched-chain alpha-keto acid dehydrogenase complex	P09062	*Pseudomonas putida*	Cytoplasm
Dihydroorotate dehydrogenase (quinone)	A5CS38	*Clavibacter michiganensis subsp. michiganensis* (strain NCPPB 382)	Plasma membrane
Enolase	Q98Q50	*Mycoplasmopsis pulmonis* (strain UAB CTIP)	Extracellular
NAD kinase	Q39JD3	*Burkholderia lata* (strain ATCC 17760 / DSM 23089 / LMG 22485 / NCIMB 9086 / R18194 / 383)	Cytoplasm

<u>DNA metabolism</u>

UvrABC system protein B	P0A8F8	*Escherichia coli* (strain K12)	Cytoplasm
DNA-3-methyladenine glycosylase 2	P04395	*Escherichia coli* (strain K12)	Cytoplasm
UvrABC system protein A	P0A698	*Escherichia coli* (strain K12)	Cytoplasm
Probable endonuclease 4	P75457	*Mycoplasma pneumoniae* (strain ATCC 29342 / M129)	Cytoplasm

UvrABC system protein C	Q5P080	*Aromatoleum aromaticum* (strain EbN1)	Cytoplasm
DNA recombination protein RmuC	P0AG71	*Escherichia coli* (strain K12)	Unknown
Prophage integrase IntA	P32053	*Escherichia coli* (strain K12)	Unknown
Holliday junction ATP-dependent DNA helicase RuvA	P75243	*Mycoplasma pneumoniae* (strain ATCC 29342 / M129)	Cytoplasm
Tyrosine recombinase XerD	Q88MV0	*Pseudomonas putida* (strain ATCC 47054 / DSM 6125 / CFBP 8728 / NCIMB 11950 / KT2440)	Cytoplasm
<u>Cell wall organization and structural maintenance</u>			
Soluble lytic murein transglycosylase	P0AGC3	*Escherichia coli* (strain K12)	Periplasmic space
D-alanyl-D-alanine carboxypeptidase DacB	P24228	*Escherichia coli* (strain K12)	Periplasmic space
Cell shape-determining protein MreB	P0A9X4	*Escherichia coli* (strain K12)	Plasma membrane
Probable L,D-transpeptidase ErfK/SrfK	P39176	*Escherichia coli* (strain K12)	Periplasmic space
Cell shape-determining protein MreC	P16926	*Escherichia coli* (strain K12)	Plasma membrane
Membrane-bound lytic murein transglycosylase A	P0A935	*Escherichia coli* (strain K12)	Plasma membrane
Murein tetrapeptide carboxypeptidase	P76008	*Escherichia coli* (strain K12)	Cytoplasm
Murein DD-endopeptidase MepH	P76190	*Escherichia coli* (strain K12)	Periplasmic space
Bifunctional protein GlmU	Q0BSR5	*Granulibacter bethesdensis* (strain ATCC BAA-1260 / CGDNIH1)	Cytoplasm
Sensor protein LytS	Q5HLG3	*Staphylococcus epidermidis* (strain ATCC 35984 / RP62A)	Plasma membrane
UDP-N-acetylglucosamine 1-carboxyvinyltransferase	A8GW63	*Rickettsia bellii (strain OSU 85-389)*	Cytoplasm
Undecaprenyl-phosphate alpha-N-acetylglucosaminyl 1-phosphate transferase	Q9X1N5	*Thermotoga maritima* (strain ATCC 43589 / DSM 3109 / JCM 10099 / NBRC 100826 / MSB8)	Plasma membrane

D-alanine--D-alanine ligase	Q3IFY2	*Pseudoalteromonas translucida* (strain TAC 125)	Cytoplasm
Inner membrane protein YdcZ	P76111	*Escherichia coli* (strain K12)	Plasma membrane
Flagellar basal-body rod protein FlgG	P24500	*Bacillus subtilis* (strain 168)	Unknown
Flagellin	Q06968	*Salmonella berta*	Extracellular
Cell division protein FtsZ	P0A9A6	*Escherichia coli* (strain K12)	Plasma membrane
Probable septum site-determining protein MinC	Q63RS4	*Burkholderia pseudomallei* (strain K96243)	Cytoplasm
Cytadherence high molecular weight protein 3	Q50360	*Mycoplasma pneumoniae* (strain ATCC 29342 / M129)	cytoplasm

<u>Protein Biosynthesis and Metabolism</u>

50S ribosomal protein L3	P60438	*Escherichia coli* (strain K12)	Ribosome
30S ribosomal protein S17	P0AG63	*Escherichia coli* (strain K12)	Cytoplasm
50S ribosomal protein L4	P75579	*Mycoplasma pneumoniae* (strain ATCC 29342 / M129)	Ribosome
30S ribosomal protein S2	A1TN69	*Acidovorax citrulli* (strain AAC00-1)	Cytoplasm
50S ribosomal protein L14	A9BH95	*Petrotoga mobilis* (strain DSM 10674 / SJ95)	Ribosome
30S ribosomal protein S11	A3N380	*Actinobacillus pleuropneumoniae serotype 5b* (strain L20)	Ribosome
30S ribosomal protein S5	Q5P315	*Aromatoleum aromaticum* (strain EbN1)	Small ribosomal unit
30S ribosomal protein S6	Q3JEJ6	*Nitrosococcus oceani* (strain ATCC 19707 / BCRC 17464 / JCM 30415 / NCIMB 11848 / C-107)	Ribosome
30S ribosomal protein S3	Q8R7W0	*Caldanaerobacter subterraneus subsp. tengcongensis* (strain DSM 15242 / JCM 11007 / NBRC 100824 / MB4)	Ribosome
50S ribosomal protein L28	Q5PAC9	*Anaplasma marginale* (strain St. Maries)	Ribosome

50S ribosomal protein L24	A9KJI3	*Lachnoclostridium phytofermentans* (strain ATCC 700394 / DSM 18823 / ISDg)	Ribosome
Chromophore lyase CpcS/CpeS 2	Q10XT3	*Trichodesmium erythraeum* (strain IMS101)	Cytoplasm
Aspartate aminotransferase	P00509	*Escherichia coli* (strain K12)	Cytoplasm
D-amino acid dehydrogenase	P0A6J5	*Escherichia coli* (strain K12)	Plasma membrane
Bifunctional protein TrpGD	P00904	*Escherichia coli* (strain K12)	Cytoplasm
Elongation factor P--(R)-beta-lysine ligase	B7LC16	*Escherichia coli* (strain K12)	Cytoplasm
4-aminobutyrate aminotransferase PuuE	P50457	*Escherichia coli* (strain K12)	Cytoplasm
Acetolactate synthase isozyme 1 large subunit	P08142	*Escherichia coli* (strain K12)	Cytoplasm
Cystathionine gamma-synthase	P00935	*Escherichia coli* (strain K12)	Cytoplasm
Glutamate synthase [NADPH] large chain	P09831	*Escherichia coli* (strain K12)	Cytoplasm
Glutamate decarboxylase beta	P69910	*Escherichia coli* (strain K12)	Cytoplasm
Ornithine carbamoyltransferase	A7ZVD3	*Escherichia coli O157:H7*	Cytoplasm
Aspartate aminotransferase	Q56114	*Salmonella typhi*	Cytoplasm
GMP synthase [glutamine-hydrolyzing]	A3MZV8	*Actinobacillus pleuropneumoniae serotype 5b* (strain L20)	Cytoplasm
Chorismate synthase	Q1QQ46	*Nitrobacter hamburgensis* (strain DSM 10229 / NCIMB 13809 / X14)	Cytoplasm
3-dehydroquinate dehydratase	B4RCS3	*Phenylobacterium zucineum* (strain HLK1)	Cytoplasm
Diaminopimelate epimerase	A1SAP5	*Shewanella amazonensis* (strain ATCC BAA-1098 / SB2B)	Cytoplasm
Arginine biosynthesis bifunctional protein ArgJ	Q81M96	*Bacillus anthracis*	Cytoplasm
4-hydroxy-tetrahydrodipicolinate reductase	A9HE99	*Gluconacetobacter diazotrophicus* (strain ATCC 49037 / DSM 5601 / CCUG 37298 / CIP 103539 / LMG 7603 / PAl5)	Cytoplasm
Tryptophan synthase beta chain	Q13EQ2	*Rhodopseudomonas palustris* (strain BisB5)	Cytoplasm
Acetolactate synthase large subunit IlvB1	P9WG41	*Mycobacterium tuberculosis* (strain ATCC 25618 / H37Rv)	Cytoplasm

Glycine--tRNA ligase beta subunit	A4J7C9	*Desulfotomaculum reducens* (strain MI-1)	Cytoplasm
o-succinylbenzoate synthase	Q838J7	*Enterococcus faecalis* (strain ATCC 700802 / V583)	Cytoplasm
Ketol-acid reductoisomerase (NADP(+))	Q17X66	*Helicobacter acinonychis* (strain Sheeba)	Cytoplasm
Glycine cleavage system H protein	A3QHI1	*Shewanella loihica* (strain ATCC BAA-1088 / PV-4)	Cytoplasm
Cysteine synthase A	P0ABK6	*Escherichia coli O157:H7*	Cytoplasm
Diaminopimelate decarboxylase	Q8K9C4	*Buchnera aphidicola subsp. Schizaphis graminum* (strain Sg)	Cytoplasm
3-phosphoshikimate 1-carboxyvinyltransferase	B2U886	*Ralstonia pickettii* (strain 12J)	Cytoplasm
Histidinol-phosphate aminotransferase	B9KDN6	*Campylobacter lari* (strain RM2100 / D67 / ATCC BAA-1060)	Cytoplasm
Pyridoxal 5'-phosphate synthase subunit PdxT	Q9KGN5	*Alkalihalobacillus halodurans* (strain ATCC BAA-125 / DSM 18197 / FERM 7344 / JCM 9153 / C-125)	Cytoplasm
Kynurenine formamidase	Q2T0N2	*Burkholderia thailandensis* (strain ATCC 700388 / DSM 13276 / CIP 106301 / E264)	Cytoplasm
Cytosol non-specific dipeptidase	P15288	*Escherichia coli* (strain K12)	Cytoplasm
Dipeptidyl carboxypeptidase	P24171	*Escherichia coli* (strain K12)	Cytoplasm
ATP-dependent Clp protease proteolytic subunit	Q928C4	*Listeria innocua serovar 6a* (strain ATCC BAA-680 / CLIP 11262)	Cytoplasm
Protease HtpX homolog	B4S7I8	*rosthecochloris aestuarii* (strain DSM 271 / SK 413)	Plasma membrane
Carbamoyl dehydratase HypE	P24193	*Escherichia coli* (strain K12)	Cytoplasm
Cysteine--tRNA ligase	P75423	*Mycoplasma pneumoniae* (strain ATCC 29342 / M129)	Cytoplasm
Leucine--tRNA ligase	P75398	*Mycoplasma pneumoniae* (strain ATCC 29342 / M129)	Cytoplasm
Serine--tRNA ligase	P75107	*Mycoplasma pneumoniae* (strain ATCC 29342 / M129)	Cytoplasm

Valine--tRNA ligase	P75304	*Mycoplasma pneumoniae* (strain ATCC 29342 / M129)	Cytoplasm
Glutamyl-tRNA(Gln) amidotransferase subunit A	P75534	*Mycoplasma pneumoniae* (strain ATCC 29342 / M129)	Cytoplasm
Phenylalanine--tRNA ligase alpha subunit	Q5GTJ5	*Wolbachia sp. subsp. Brugia malayi* (strain TRS)	Cytoplasm
Valine--tRNA ligase	P36420	*Lactobacillus casei*	Cytoplasm
Proline--tRNA ligase	B8CW65	*Halothermothrix orenii* (strain H 168 / OCM 544 / DSM 9562)	Cytoplasm
Alanine--tRNA ligase	Q03YQ2	*Leuconostoc mesenteroides subsp. mesenteroides* (strain ATCC 8293 / DSM 20343 / BCRC 11652 / CCM 1803 / JCM 6124 / NCDO 523 / NBRC 100496 / NCIMB 8023 / NCTC 12954 / NRRL B-1118 / 37Y)	Cytoplasm
Threonine--tRNA ligase	B0TT20	*Shewanella halifaxensis* (strain HAW-EB4)	Cytoplasm
Peptide chain release factor 1	B8HB03	*Pseudarthrobacter chlorophenolicus* (strain ATCC 700700 / DSM 12829 / CIP 107037 / JCM 12360 / KCTC 9906 / NCIMB 13794 / A6)	Cytoplasm
Regulation Factor and RNA Processing			
Multifunctional CCA protein	P06961	*Escherichia coli* (strain K12)	Cytoplasm
Ribosomal RNA small subunit methyltransferase D	P0ADX9	*Escherichia coli* (strain K12)	Cytoplasm
Ribonuclease 3	P0A7Y0	*Escherichia coli* (strain K12)	Cytoplasm
tRNA threonylcarbamoyladenosine dehydratase	Q46927	*Escherichia coli* (strain K12)	Plasma membrane
tRNA-2-methylthio-N(6)-dimethylallyladenosine synthase	P0AEI1	*Escherichia coli* (strain K12)	Cytoplasm

Putative pre-16S rRNA nuclease	P57114	*Mycoplasma pneumoniae* (strain ATCC 29342 / M129)	Cytoplasm
Ribonuclease Y	P75506	*Mycoplasma pneumoniae* (strain ATCC 29342 / M129)	Plasma membrane
tRNA-specific 2-thiouridylase MnmA	A7I2L9	*Campylobacter hominis* (strain ATCC BAA-381 / LMG 19568 / NCTC 13146 / CH001A)	Cytoplasm
Ribosomal RNA small subunit methyltransferase A	A4YJT3	*Bradyrhizobium sp.* (strain BTAi1 / ATCC BAA-1182)	Cytoplasm
Ribosomal protein S12 methylthiotransferase RimO	Q7VKK2	*Haemophilus ducreyi* (strain 35000HP / ATCC 700724)	Cytoplasm
Probable RNA 2'-phosphotransferase	Q395F2	*Burkholderia lata* (strain ATCC 17760 / DSM 23089 / LMG 22485 / NCIMB 9086 / R18194 / 383)	Cytoplasm
tRNA uridine (34) hydroxylase	Q7UFS5	*Rhodopirellula baltica* (strain DSM 10527 / NCIMB 13988 / SH1)	Cytoplasm
tRNA modification GTPase MnmE	A5VA82	*Rhizorhabdus wittichii* (strain DSM 6014 / CCUG 31198 / JCM 15750 / NBRC 105917 / EY 4224 / RW1)	Cytoplasm
Elongation factor Ts	A8MHH0	*Alkaliphilus oremlandii* (strain OhILAs)	Cytoplasm
Ribosomal RNA large subunit methyltransferase H	Q2NUV2	*Sodalis glossinidius* (strain morsitans)	Cytoplasm
Ribonuclease Z	A6LU82	*Clostridium beijerinckii* (strain ATCC 51743 / NCIMB 8052)	Cytoplasm
Elongation factor Tu	A7HBL7	*Anaeromyxobacter sp.* (strain Fw109-5)	Cytoplasm
GTPase Der	A1SU43	*Psychromonas ingrahamii* (strain 37)	Cytoplasm
Elongation factor G 2	Q3IJW9	*Pseudoalteromonas translucida* (strain TAC 125)	Cytoplasm

Regulation of transcription

Uxu operon transcriptional regulator	P39161	*Escherichia coli* (strain K12)	Cytoplasm
HTH-type transcriptional regulator ZntR	P0ACS5	*Escherichia coli* (strain K12)	Cytoplasm
Psp operon transcriptional activator	P37344	*Escherichia coli* (strain K12)	Cytoplasm
Propionate catabolism operon regulatory protein	P77743	*Escherichia coli* (strain K12)	Cytoplasm
Sugar fermentation stimulation protein B	P0ACH1	*Escherichia coli* (strain K12)	Unknown
DNA polymerase I	P00582	*Escherichia coli* (strain K12)	Cytoplasm
OriC-binding nucleoid-associated protein	P64467	*Escherichia coli* (strain K12)	Cytoplasm
DNA-directed RNA polymerase subunit alpha	Q50295	*Mycoplasma pneumoniae* (strain ATCC 29342 / M129)	Cytoplasm
Biofilm growth-associated repressor	Q8UAA8	*Agrobacterium fabrum* (strain C58 / ATCC 33970)	Cytoplasm
Transcriptional regulatory protein PrrA	Q50136	*Mycobacterium leprae* (strain TN)	Cytoplasm

DNA replication

Beta sliding clamp	P0A988	*Escherichia coli* (strain K12)	Cytoplasm
Anaerobic ribonucleoside-triphosphate reductase	P28903	*Escherichia coli* (strain K12)	Cytoplasm
Ribonucleoside-diphosphate reductase subunit alpha	P78027	*Mycoplasma pneumoniae* (strain ATCC 29342 / M129)	Cytoplasm
LexA repressor	O33927	*Thermotoga maritima* (strain ATCC 43589 / DSM 3109 / JCM 10099 / NBRC 100826 / MSB8)	Cytoplasm
Flap endonuclease Xni	A1S8B8	*Shewanella amazonensis (strain ATCC BAA-1098 / SB2B)*	Cytoplasm

Chromosomal replication initiator protein DnaA	Q1MSG8	*Lawsonia intracellularis* (strain PHE/MN1-00)	Cytoplasm

Cell signaling

Signal recognition particle protein	P0AGD7	*Escherichia coli* (strain K12)	Ribosome
Bacteriophage adsorption protein A	P31600	*Escherichia coli* (strain K12)	Cell outer membrane
Sensor protein EvgS	P30855	*Escherichia coli* (strain K12)	Plasma membrane
Sensor histidine kinase EnvZ	P0AEJ4	*Escherichia coli* (strain K12)	Plasma membrane

Ion assimilation

Ferric iron reductase protein FhuF	P39405	*Escherichia coli* (strain K12)	Plasma membrane
Periplasmic nitrate reductase	P33937	*Escherichia coli* (strain K12)	Periplasmic space
Anaerobic nitric oxide reductase flavorubredoxin	A0KEJ1	*Aeromonas hydrophila subsp. hydrophila* (strain ATCC 7966 / DSM 30187 / BCRC 13018 / CCUG 14551 / JCM 1027 / KCTC 2358 / NCIMB 9240 / NCTC 8049)	Cytoplasm

Transferase

3-oxoadipyl-CoA/3-oxo-5,6-dehydrosuberyl-CoA thiolase	P0C7L2	*Escherichia coli* (strain K12)	Cytoplasm
Trehalose-6-phosphate synthase	P31677	*Escherichia coli* (strain K12)	Cytoplasm
Propionyl-CoA:succinate CoA transferase	P52043	*Escherichia coli* (strain K12)	Cytoplasm
Putative phosphotransferase enzyme IIB component MPN_268	P75507	*Mycoplasma pneumoniae* (strain ATCC 29342 / M129)	Plasma membrane

Ubiquinone biosynthesis O-methyltransferase	A8LQ43	*Dinoroseobacter shibae* (strain DSM 16493 / NCIMB 14021 / DFL 12)	Cytoplasm
tRNA-cytidine (32) 2-sulfurtransferase	A4G9W3	*Herminiimonas arsenicoxydans*	Cytoplasm
Probable transaldolase	A5GBY8	*Geotalea uraniireducens* (strain Rf4)	Cytoplasm
Nucleotide metabolism			
Nucleoside diphosphate kinase	A8AD67	*Citrobacter koseri* (strain ATCC BAA-895 / CDC 4225-83 / SGSC4696)	Cytoplasm
Probable dihydroorotate dehydrogenase A (fumarate)	P59626	*Enterococcus faecalis* (strain ATCC 700802 / V583)	Cytoplasm
Uridine phosphorylase	O08444	*Klebsiella aerogenes*	Cytoplasm
Thymidylate kinase	C4KZW9	*Exiguobacterium sp.* (strain ATCC BAA-1283 / AT1b)	Cytoplasm
Thymidylate synthase	A7MXJ9	*Vibrio campbellii* (strain ATCC BAA-1116 / BB120)	Cytoplasm
Adenosine deaminase	P22333	*Escherichia coli* (strain K12)	Cytoplasm
Allantoinase	Q9RV76	*Deinococcus radiodurans* (strain ATCC 13939 / DSM 20539 / JCM 16871 / LMG 4051 / NBRC 15346 / NCIMB 9279 / R1 / VKM B-1422)	Cytoplasm
Putative carbamate hydrolase RutD	Q32HQ2	*Shigella dysenteriae serotype 1* (strain Sd197)	Unknown
Dihydroorotate dehydrogenase B (NAD(+))	Q8EUY2	*Mycoplasma penetrans* (strain HF-2)	Cytoplasm
Probable glycine dehydrogenase (decarboxylating) subunit 1	B5Y9D4	*Coprothermobacter proteolyticus* (strain ATCC 35245 / DSM 5265 / OCM 4 / BT)	Cytoplasm
Transmembrane transporters			
Protein TonB	P02929	*Escherichia coli* (strain K12)	Periplasmic space

Sodium/pantothenate symporter	P16256	*Escherichia coli* (strain K12)	Plasma membrane
Proline-specific permease ProY	P0AAE2	*Escherichia coli* (strain K12)	Plasma membrane
D-allose import ATP-binding protein AlsA	P32721	*Escherichia coli* (strain K12)	Plasma membrane
Glutamate/aspartate import ATP-binding protein GltL	P0AAG3	*Escherichia coli* (strain K12)	Plasma membrane
L-cystine-binding protein TcyJ	P0AEM9	*Escherichia coli* (strain K12)	Plasma membrane
Inner membrane metabolite transport protein YdjE	P38055	*Escherichia coli* (strain K12)	Plasma membrane
Outer membrane porin C	P06996	*Escherichia coli* (strain K12)	Cell outer membrane
Miniconductance mechanosensitive channel YbdG	P0AAT4	*Escherichia coli* (strain K12)	Plasma membrane
Polyamine export protein	P0AE45	*Escherichia coli* (strain K12)	Cell outer membrane
Putative nucleoside permease NupX	P33021	*Escherichia coli* (strain K12)	Plasma membrane
AI-2 transport protein TqsA	P0AFS5	*Escherichia coli* (strain K12)	Cell inner membrane
Multiphosphoryl transfer protein	P32670	*Escherichia coli* (strain K12)	Cytoplasm
Flagellar biosynthetic protein FlhB	P76299	*Escherichia coli* (strain K12)	Plasma membrane
p-hydroxybenzoic acid efflux pump subunit AaeA	P46481	*Escherichia coli* (strain K12)	Plasma membrane
Nitrate/nitrite transporter NarU	P37758	*Escherichia coli* (strain K12)	Plasma membrane
Phosphate import ATP-binding protein PstB	P75186	*Mycoplasma pneumoniae* (strain ATCC 29342 / M129)	Plasma membrane
Methionine import ATP-binding protein MetN	Q8RFN2	*Fusobacterium nucleatum subsp. nucleatum* (strain ATCC 25586 / DSM 15643 / BCRC 10681 / CIP 101130 / JCM 8532 / KCTC 2640 / LMG 13131 / VPI 4355)	Plasma membrane
Probable TonB-dependent receptor NMB0964	Q9JZN9	*Neisseria meningitidis serogroup B* (strain MC58)	Plasma membrane
Probable membrane transporter protein YdhB	O05493	*Bacillus subtilis* (strain 168)	Plasma membrane
Putative multidrug export ATP-binding/permease protein SA1683	Q7A4T3	*Staphylococcus aureus* (strain N315)	Plasma membrane
Protein translocase subunit SecA	Q9K6W8	*Alkalihalobacillus halodurans* (strain ATCC BAA-125 / DSM 18197 / FERM 7344 / JCM 9153 / C-125)	Cytoplasm
C4-dicarboxylate-binding periplasmic protein DctP	Q9HU18	*Pseudomonas aeruginosa* (strain ATCC 15692 / DSM	Periplasmic space

		22644 / CIP 104116 / JCM 14847 / LMG 12228 / 1C / PRS 101 / PAO1)	
Isoprene biosynthesis			
4-hydroxy-3-methylbut-2-en-1-yl diphosphate synthase (flavodoxin)	Q6G104	*Bartonella quintana* (strain Toulouse)	Cytoplasm
Bifunctional enzyme IspD/IspF	A5V2U9	*Rhizorhabdus wittichii* (strain DSM 6014 / CCUG 31198 / JCM 15750 / NBRC 105917 / EY 4224 / RW1)	Cytoplasm
Light dependent process			
Light-independent protochlorophyllide reductase subunit B	Q132N3	*Rhodopseudomonas palustris* (strain BisB5)	Cytoplasm
Protein Thf1	Q8YZ41	*Nostoc sp.* (strain PCC 7120 / SAG 25.82 / UTEX 2576)	Cytoplasm
Pathogenesis			
Cytolysin RtxA	A1YKW7	*Kingella kingae*	Extracellular
Putative colanic acid biosynthesis glycosyl transferase WcaI	P32057	*Escherichia coli* (strain K12)	Cytoplasm
Lipopolysaccharide 1,2-N-acetylglucosaminetransferase	P27242	*Escherichia coli* (strain K12)	Cytoplasm
Unknown			
Oxidoreductase UcpA	P37440	*Escherichia coli* (strain K12)	Cytoplasm
Putative inactive recombination-promoting nuclease-like protein YjiQ	P0DP22	*Escherichia coli* (strain K12)	Cytoplasm
Lipoprotein YfjS	O52982	*Escherichia coli* (strain K12)	Plasma membrane

Protein YhfA	P0ADX1	*Escherichia coli* (strain K12)	Cytoplasm
Hydrogenase-4 component I	P77668	*Escherichia coli* (strain K12)	Plasma membrane
Probable ATP-dependent helicase lhr	P30015	*Escherichia coli* (strain K12)	Cytoplasm
Hydrogenase-4 component J	P77453	*Escherichia coli* (strain K12)	Plasma membrane
Insertion element IS150 protein InsJ	P19768	*Escherichia coli* (strain K12)	Cytoplasm
Protein YrdA	P0A9W9	*Escherichia coli* (strain K12)	Cytoplasm
Probable cyclic di-GMP phosphodiesterase PdeI	P75800	*Escherichia coli* (strain K12)	Plasma membrane
Probable ATP-dependent RNA helicase MG425 homolog	P75172	*Mycoplasma pneumoniae* (strain ATCC 29342 / M129)	Cytoplasm
Putative adhesin P1-like protein MPN_370	P75411	*Mycoplasma pneumoniae* (strain ATCC 29342 / M129)	Extracellular
UPF0134 protein MPN_675	P75117	*Mycoplasma pneumoniae* (strain ATCC 29342 / M129)	Unknown
Translation initiation factor IF-2	C5CDZ4	*Kosmotoga olearia* (strain ATCC BAA-1733 / DSM 21960 / TBF 19.5.1)	Cytoplasm
NAD(P)H dehydrogenase (quinone)	B9M9X5	*Acidovorax ebreus* (strain TPSY)	Unknown
General stress protein 26	P80238	*Bacillus subtilis* (strain 168)	Unknown
7,8-didemethyl-8-hydroxy-5-deazariboflavin synthase	P73191	*Synechocystis sp.* (strain PCC 6803 / Kazusa)	Cytoplasm
UPF0102 protein Sama_3355	A1SB01	*Shewanella amazonensis* (strain ATCC BAA-1098 / SB2B)	Cytoplasm
Oligoribonuclease	Q2L2P5	*Bordetella avium* (strain 197N)	Cytoplasm
UPF0229 protein Meso_0256	Q11LR5	*Chelativorans sp.* (strain BNC1)	Cytoplasm
Quinone-reactive Ni/Fe-hydrogenase large chain	P31883	*Wolinella succinogenes* (strain ATCC 29543 / DSM 1740 / LMG 7466 / NCTC 11488 / FDC 602W)	Plasma membrane
Lipoprotein LpqB	Q47LZ	*Thermobifida fusca* (strain YX)	Plasma membrane

Supplementary Table 2 – DMSO group unique proteins identified by ESI-LC-MS/MS

Protein Name	ID uniprot	Organism Reference	Cellular Compartment
**<u>Carbohydrate metabolism</u>**			
Maltodextrin phosphorylase	P00490	*Escherichia coli* (strain K12)	Cytoplasm
L-fuculokinase	P11553	*Escherichia coli* (strain K12)	Cytoplasm
Hexitol phosphatase B	P77247	*Escherichia coli* (strain K12)	Cytoplasm
Putative beta-xylosidase	P77713	*Escherichia coli* (strain K12)	Unknown
Lactaldehyde dehydrogenase	P25553	*Escherichia coli* (strain K12)	Cytoplasm
3-oxo-tetronate kinase	Q46889	*Escherichia coli* (strain K12)	Unknown
dTDP-4-amino-4,6-dideoxygalactose transaminase	P27833	*Escherichia coli* (strain K12)	Cytoplasm
Sulfoquinovose isomerase	P32140	*Escherichia coli* (strain K12)	Cytoplasm
Putative 1-phosphofructokinase	P75038	*Mycoplasma pneumoniae (strain ATCC 29342 / M129)*	Plasma membrane
Transaldolase	Q66ET5	*Yersinia pseudotuberculosis serotype I* (strain IP32953)	Cytoplasm
5-oxoprolinase subunit A	A0JR24	*Arthrobacter sp.* (strain FB24)	Unknown
<u>Cell Redox Homeostasis</u>			
Alkyl hydroperoxide reductase C	P0AE08	*Escherichia coli* (strain K12)	Cytoplasm
Alkyl hydroperoxide reductase subunit F	P35340	*Escherichia coli* (strain K12)	Cytoplasm
Probable NADH oxidase	P75389	*Mycoplasma pneumoniae* (strain ATCC 29342 / M129)	Cytoplasm

<u>Stress and Defense Response</u>

ATP-dependent RNA helicase DeaD	P0A9P6	*Escherichia coli* (strain K12)	Periplasmic space
UDP-4-amino-4-deoxy-L-arabinose--oxoglutarate aminotransferase	P77690	*Escherichia coli (strain K12)*	Cytoplasm
Multidrug resistance protein MdtN	P32716	*Escherichia coli (strain K12)*	Plasma membrane
UPF0194 membrane protein YbhG	P75777	*Escherichia coli (strain K12)*	Plasma membrane
Probable multidrug resistance protein EmrK	P52599	*Escherichia coli (strain K12)*	Plasma membrane
Chaperone protein ClpB	Q88VX7	*Escherichia coli (strain K12)*	Cytoplasm
Protein GrpE	P78017	*Mycoplasma pneumoniae* (strain ATCC 29342 / M129)	Cytoplasm
Chaperonin GroEL	P78012	*Mycoplasma pneumoniae* (strain ATCC 29342 / M129)	Cytoplasm
Co-chaperonin GroES	A7ZCV1	*Campylobacter concisus* (strain 13826)	Cytoplasm
Chaperone protein DnaK	C4K3I6	*Hamiltonella defensa subsp. Acyrthosiphon pisum* (strain 5AT)	Cytoplasm
Chaperone protein ClpB	Q88VX7	*Lactiplantibacillus plantarum* (strain ATCC BAA-793 / NCIMB 8826 / WCFS1)	Cytoplasm

Energy and Metabolism

Pyruvate dehydrogenase E1 component	P0AFG8	*Escherichia coli* (strain K12)	Cytoplasm
Phosphoglucomutase	P36938	*Escherichia coli* (strain K12)	Cytoplasm
Glucose-6-phosphate isomerase	P0A6T1	*Escherichia coli* (strain K12)	Cytoplasm
Acyl carrier protein	P0A6A8	*Escherichia coli* (strain K12)	Cytoplasm
Citrate synthase	P0ABH7	*Escherichia coli* (strain K12)	Cytoplasm
Cytochrome bo(3) ubiquinol oxidase subunit 1	P0ABI8	*Escherichia coli* (strain K12)	Plasma membrane
Phosphoglycerate kinase	P0A799	*Escherichia coli* (strain K12)	Cytoplasm
Formate hydrogenlyase subunit 7 dehydrogenase C2	P16433	*Escherichia coli* (strain K12)	Cytoplasm
Glutathione-specific gamma-glutamylcyclotransferase	P39163	*Escherichia coli* (strain K12)	Cytoplasm

NADH-quinone oxidoreductase subunit I	P0AFD6	*Escherichia coli* (strain K12)	Plasma membrane
Galactokinase	P0A6T3	*Escherichia coli* (strain K12)	Cytoplasm
Fructose-bisphosphate aldolase	P75089	*Mycoplasma pneumoniae* (strain ATCC 29342 / M129)	Cytoplasm
Enolase	P75189	*Mycoplasma pneumoniae* (strain ATCC 29342 / M129)	Cytoplasm
Acetate kinase	P75245	*Mycoplasma pneumoniae* (strain ATCC 29342 / M129)	Cytoplasm
Phosphoglycerate kinase	A7MJQ4	*Cronobacter sakazakii* (strain ATCC BAA-894)	Cytoplasm
Adenylate kinase	A5FZU4	*Acidiphilium cryptum* (strain JF-5)	Cytoplasm
Glyceraldehyde-3-phosphate dehydrogenase	Q07234	*Buchnera aphidicola subsp. Schizaphis graminum* (strain Sg)	Cytoplasm
Phosphoenolpyruvate synthase	P23538	*Escherichia coli* (strain K12)	Cytoplasm
methylglyoxal synthase	P0A731	*Escherichia coli* (strain K12)	Cytoplasm
Phosphoribosyl 1,2-cyclic phosphate phosphodiesterase	P16692	*Escherichia coli* (strain K12)	Cytoplasm
Malate dehydrogenase	B1XHK9	*Escherichia coli* (strain K12)	Periplasmic space
Phosphoenolpyruvate carboxylase	A1U2U4	*Marinobacter nauticus* (strain ATCC 700491 / DSM 11845 / VT8)	Cytoplasm
Dihydroorotate dehydrogenase (quinone)	Q8DLB7	*Thermosynechococcus vestitus* (strain IAM M-273 / NIES-2133 / BP-1)	Cytoplasm
Glycerol kinase	B9JZR4	*Agrobacterium vitis* (strain S4 / ATCC BAA-846)	Cytoplasm
NADPH-Fe(3+) oxidoreductase subunit alpha	Q74FU6	*Geobacter sulfurreducens* (strain ATCC 51573 / DSM 12127 / PCA)	Cytoplasm
ATP synthase subunit delta	A6H2D8	*Flavobacterium psychrophilum* (strain ATCC 49511 / DSM 21280 / CIP 103535 / JIP02/86)	Plasma membrane
Glucokinase	C5BCK8	*Edwardsiella ictaluri* (strain 93-146)	Cytoplasm

1,4-alpha-glucan branching enzyme GlgB	Q04KG8	*Streptococcus pneumoniae serotype 2* (strain D39 / NCTC 7466)	Cytoplasm
PTS system glucose-specific EIICBA component	P20166	*Bacillus subtilis* (strain 168)	Plasma membrane
Phosphoglycerate kinase	Q47XD4	*Colwellia psychrerythraea* (strain 34H / ATCC BAA-681)	Cytoplasm
Succinate--CoA ligase [ADP-forming] subunit beta	A4W879	*Enterobacter sp.* (strain 638)	Cytoplasm
ATP synthase gamma chain	Q3IK49	*Pseudoalteromonas translucida* (strain TAC 125)	Plasma membrane

DNA metabolism

DNA base-flipping protein	P0AFP2	*Escherichia coli (strain K12)*	Unknown
Transcription-repair-coupling factor	P30958	*Escherichia coli (strain K12)*	Cytoplasm
Exodeoxyribonuclease I	Q2A0L0	*Escherichia coli (strain K12)*	Cytoplasm
Formamidopyrimidine-DNA glycosylase	P42371	*Lactococcus lactis subsp. cremoris*	Cytoplasm
DNA repair protein RecN	P17894	*Bacillus subtilis (strain 168)*	Cytoplasm
DNA mismatch repair protein MutS	Q38YR4	*Latilactobacillus sakei subsp. sakei* (strain 23K)	Cytoplasm
Protein RecA	Q05358	*Legionella pneumophila*	Cytoplasm
DNA utilization protein HofM	P45753	*Escherichia coli* (strain K12)	Unknown
RecBCD enzyme subunit RecB	P08394	*Escherichia coli* (strain K12)	Cytoplasm
5'-3' exonuclease	P75403	Mycoplasma pneumoniae (strain ATCC 29342 / M129)	Cytoplasm
Exodeoxyribonuclease 7 large subunit	Q2RIB5	*Moorella thermoacetica* (strain ATCC 39073 / JCM 9320)	Cytoplasm
Crossover junction endodeoxyribonuclease RuvC	Q820F5	*Streptomyces avermitilis* (strain ATCC 31267 / DSM 46492 / JCM 5070 / NBRC 14893 / NCIMB 12804 / NRRL 8165 / MA-4680)	Cytoplasm

Cell signaling

Signal recognition particle receptor FtsY	P75362	*Mycoplasma pneumoniae* (strain ATCC 29342 / M129)	Plasma membrane

Methyl-accepting chemotaxis protein I	P02942	*Escherichia coli* (strain K12)	Cell inner membrane

Protein Biosynthesis and Metabolism

50S ribosomal protein L14	P0ADY3	*Escherichia coli* (strain K12)	Cytoplasm
50S ribosomal protein L27	P0ABI8	*Escherichia coli* (strain K12)	Cytoplasm
Protein NrdI	P0A772	*Escherichia coli* (strain K12)	Unknown
50S ribosomal protein L16	P41204	*Mycoplasma pneumoniae* (strain ATCC 29342 / M129)	Cytoplasm
50S ribosomal protein L17	Q59547	*Mycoplasma pneumoniae* (strain ATCC 29342 / M129)	Ribosome
50S ribosomal protein L22	A8AQL2	*Citrobacter koseri* (strain ATCC BAA-895 / CDC 4225-83 / SGSC4696)	Ribosome
50S ribosomal protein L29	Q6A6M8	*Cutibacterium acnes* (strain DSM 16379 / KPA171202)	Ribosome
50S ribosomal protein L22	Q3KLH4	*Chlamydia trachomatis serovar A* (strain ATCC VR-571B / DSM 19440 / HAR-13)	Ribosome
Pup--protein ligase	D1BS28	*Xylanimonas cellulosilytica* (strain DSM 15894 / CECT 5975 / LMG 20990 / XIL07)	Cytoplasm
50S ribosomal protein L6	Q88XX1	*Lactiplantibacillus plantarum* (strain ATCC BAA-793 / NCIMB 8826 / WCFS1)	Ribosome
50S ribosomal protein L30	B9K8A4	*Thermotoga neapolitana* (strain ATCC 49049 / DSM 4359 / NBRC 107923 / NS-E)	Ribosome
30S ribosomal protein S11	Q9PJN3	*Chlamydia muridarum* (strain MoPn / Nigg)	Ribosome
Ribosome-recycling factor	C3MBQ5	*Sinorhizobium fredii* (strain NBRC 101917 / NGR234)	Cytoplasm
LexA repressor	B8HG97	*Pseudarthrobacter chlorophenolicus* (strain ATCC 700700 / DSM 12829 / CIP	Cytoplasm

		107037 / JCM 12360 / KCTC 9906 / NCIMB 13794 / A6)	
50S ribosomal protein L15	Q6CZY9	*Pectobacterium atrosepticum* (strain SCRI 1043 / ATCC BAA-672)	Ribosome
Protein translocase subunit SecA	Q8K9C4	*Buchnera aphidicola subsp. Schizaphis graminum* (strain Sg)	Plasma membrane
30S ribosomal protein S4	B0C9F5	*Acaryochloris marina* (strain MBIC 11017)	Ribosome
30S ribosomal protein S11	B3WAJ3	*Lacticaseibacillus casei* (strain BL23)	Ribosome
5,10-methylenetetrahydrofolate reductase	P0AEZ1	*Escherichia coli* (strain K12)	Cytoplasm
S-adenosylmethionine synthase	B7UHY9	*Escherichia coli* (strain K12)	Cytoplasm
D-cysteine desulfhydrase	P76316	*Escherichia coli* (strain K12)	Cytoplasm
Putative aminodehydroquinate synthase	Q3J830	*Amycolatopsis mediterranei* (strain S699)	Cytoplasm
Glutamate mutase epsilon subunit	Q05509	*Clostridium tetanomorphum*	Cytoplasm
N-acetyl-gamma-glutamyl-phosphate reductase	A5WH78	*Psychrobacter sp.* (strain PRwf-1)	Cytoplasm
Tyrosine phenol-lyase	Q9CMK9	*Pasteurella multocida* (strain Pm70)	Cytoplasm
3-dehydroquinate synthase	A3PC83	*Prochlorococcus marinus* (strain MIT 9301)	Cytoplasm
3-isopropylmalate dehydratase large subunit	Q7VAV9	*Prochlorococcus marinus* (strain SARG / CCMP1375 / SS120)	Cytoplasm
Phosphoribosyl-ATP pyrophosphatase	B1M0Q0	*Methylobacterium radiotolerans* (strain ATCC 27329 / DSM 1819 / JCM 2831 / NBRC 15690 / NCIMB 10815 / 0-1)	Cytoplasm
1D-myo-inositol 2-acetamido-2-deoxy-alpha-D-glucopyranoside deacetylase	D6Y7M2	*Thermobispora bispora* (strain ATCC 19993 / DSM 43833 / CBS 139.67 / JCM 10125 / KCTC 9307 / NBRC 14880 / R51)	Cytoplasm

Chorismate synthase	Q0S0N2	*Rhodococcus jostii* (strain RHA1)	Cytoplasm
3-phosphoshikimate 1-carboxyvinyltransferase	B9MQK4	*Caldicellulosiruptor bescii* (strain ATCC BAA-1888 / DSM 6725 / Z-1320)	Cytoplasm
Glycine cleavage system H protein	A1T9T8	*Mycolicibacterium vanbaalenii* (strain DSM 7251 / JCM 13017 / BCRC 16820 / KCTC 9966 / NRRL B-24157 / PYR-1)	Cytoplasm
Selenide, water dikinase	C4ZDB0	*Agathobacter rectalis* (strain ATCC 33656 / DSM 3377 / JCM 17463 / KCTC 5835 / VPI 0990)	Cytoplasm
Serine hydroxymethyltransferase	A6VXM6	*Marinomonas sp.* (strain MWYL1)	Cytoplasm
Imidazole glycerol phosphate synthase subunit HisH	P60601	*Rhodopseudomonas palustris* (strain ATCC BAA-98 / CGA009)	Cytoplasm
Glutamine synthetase	P10583	*Azospirillum brasilense*	Cytoplasm
Arginine biosynthesis bifunctional protein ArgJ	Q67KC5	*Symbiobacterium thermophilum* (strain T / IAM 14863)	Cytoplasm
3-phosphoshikimate 1-carboxyvinyltransferase	B8D9A1	*Buchnera aphidicola subsp. Acyrthosiphon pisum* (strain 5A)	Cytoplasm
Dihydroxy-acid dehydratase	Q2SA20	*Hahella chejuensis* (strain KCTC 2396)	Cytoplasm
Carbamoyl-phosphate synthase small chain	B2URJ0	*Akkermansia muciniphila* (strain ATCC BAA-835 / DSM 22959 / JCM 33894 / BCRC 81048 / CCUG 64013 / CIP 107961 / Muc)	Cytoplasm
Phosphoribosyl-AMP cyclohydrolase	P62392	*Leptospira interrogans serogroup Icterohaemorrhagiae serovar copenhageni* (strain Fiocruz L1-130)	Cytoplasm
Argininosuccinate synthase	Q63U95	*Burkholderia pseudomallei* (strain K96243)	Cytoplasm

Glycine--tRNA ligase beta subunit	P00961	*Escherichia coli* (strain K12)	Cytoplasm
Phosphoenolpyruvate-dependent phosphotransferase system	P37177	*Escherichia coli* (strain K12)	Cytoplasm
Methionyl-tRNA formyltransferase	P75235	*Mycoplasma pneumoniae* (strain ATCC 29342 / M129)	Cytoplasm
GTPase Era	Q97JI5	*Clostridium acetobutylicum* (strain ATCC 824 / DSM 792 / JCM 1419 / LMG 5710 / VKM B-1787)	Plasma membrane
<u>Regulation Factor and RNA Processing</u>			
Ribosomal RNA large subunit methyltransferase K/L	P0A8I8	*Escherichia coli* (strain K12)	Cytoplasm
tRNA-modifying protein YgfZ	P75810	*Escherichia coli* (strain K12)	Cytoplasm
RNA 2'-phosphotransferase	P39380	*Escherichia coli* (strain K12)	Unknown
Ribonuclease J	P75497	*Mycoplasma pneumoniae* (strain ATCC 29342 / M129)	Cytoplasm
Elongation factor Tu 1	P0CE47	*Escherichia coli O139:H28* (strain E24377A / ETEC)	Cytoplasm
Elongation factor G	Q492B1	*Blochmannia pennsylvanicus* (strain BPEN)	Cytoplasm
Phenylalanine--tRNA ligase alpha subunit	Q88WM8	*Lactiplantibacillus plantarum* (strain ATCC BAA-793 / NCIMB 8826 / WCFS1)	Cytoplasm
Phenylalanine--tRNA ligase beta subunit	P59505	*Buchnera aphidicola subsp. Baizongia pistaciae* (strain Bp)	Cytoplasm
Elongation factor 4	C3K6G8	*Pseudomonas fluorescens* (strain SBW25)	Cytoplasm
tRNA modification GTPase MnmE	Q1MPF1	*Lawsonia intracellularis* (strain PHE/MN1-00)	Cytoplasm
<u>Transferase</u>			
L-carnitine CoA-transferase	P31572	*Escherichia coli* (strain K12)	Cytoplasm
Phosphate acetyltransferase EutD	P77218	*Escherichia coli* (strain K12)	Cytoplasm
Protein adenylyltransferase SelO	Q0THC2	*Escherichia coli* (strain K12)	Unknown
3-methyl-2-oxobutanoate hydroxymethyltransferase	P31057	*Escherichia coli* (strain K12)	Cytoplasm

Nicotinate-nucleotide--dimethylbenzimidazole phosphoribosyltransferase	P36562	*Escherichia coli* (strain K12)	Cytoplasm
Putative acetyltransferase MPN_114	P75448	*Mycoplasma pneumoniae* (strain ATCC 29342 / M129)	Cytoplasm
Ornithine carbamoyltransferase	Q93JF1	*Streptomyces coelicolor* (strain ATCC BAA-471 / A3(2) / M145)	Cytoplasm
Sulfate adenylyltransferase	A5D5R7	*Pelotomaculum thermopropionicum* (strain DSM 13744 / JCM 10971 / SI)	Cytoplasm
4-diphosphocytidyl-2-C-methyl-D-erythritol kinase	Q1LTH3	*Baumannia cicadellinicola subsp. Homalodisca coagulata*	Unknown
1-deoxy-D-xylulose-5-phosphate synthase	A3MYS9	*Actinobacillus pleuropneumoniae serotype 5b* (strain L20)	Cytoplasm
Phosphatidylglycerol--prolipoprotein diacylglyceryl transferase	Q8NNU2	*Corynebacterium glutamicum* (strain ATCC 13032 / DSM 20300 / BCRC 11384 / JCM 1318 / LMG 3730 / NCIMB 10025)	Cytoplasm
Light dependent process			
Light-independent protochlorophyllide reductase subunit N	Q7V2D5	*Prochlorococcus marinus subsp. pastoris* (strain CCMP1986 / NIES-2087 / MED4)	Cytoplasm
Glutamyl-tRNA reductase	A5UU33	*Roseiflexus sp.* (strain RS-1)	Cytoplasm
Transmembrane transporters			
p-hydroxybenzoic acid efflux pump subunit AaeB	P46481	*Escherichia coli* (strain K12)	Plasma membrane
D-galactose-binding periplasmic protein	P0AEE5	*Escherichia coli* (strain K12)	Cytoplasm
Inner membrane transport protein RhmT	P76470	*Escherichia coli* (strain K12)	Plasma membrane

Putative outer membrane porin protein NmpC	P21420	*Escherichia coli* (strain K12)	Cell outer membrane
Thiosulfate-binding protein	P16700	*Escherichia coli* (strain K12)	Plasma membrane
Oligopeptide transport ATP-binding protein OppF	P75551	Mycoplasma pneumoniae (strain ATCC 29342 / M129)	Plasma membrane
Spermidine/putrescine import ATP-binding protein PotA	P75059	Mycoplasma pneumoniae (strain ATCC 29342 / M129)	Plasma membrane
Phosphate import ATP-binding protein PstB 1	Q8RCU0	*Caldanaerobacter subterraneus subsp. tengcongensis* (strain DSM 15242 / JCM 11007 / NBRC 100824 / MB4)	Plasma membrane
Fe(3+) ions import ATP-binding protein FbpC 2	Q6D2F6	*Pectobacterium atrosepticum* (strain SCRI 1043 / ATCC BAA-672)	Plasma membrane
<u>DNA replication</u>			
DNA adenine methylase	P0AEE8	*Escherichia coli* (strain K12)	Periplasmic space
50S ribosomal protein L2	P60422	*Escherichia coli* (strain K12)	Cytoplasm
Probable DNA helicase II homolog	P75437	Mycoplasma pneumoniae (strain ATCC 29342 / M129)	Cytoplasm
<u>Regulation of transcription</u>			
Arabinose operon regulatory protein	P0A9E0	*Escherichia coli* (strain K12)	Cytoplasm
HTH-type transcriptional regulator GadX	P37639	*Escherichia coli* (strain K12)	Cytoplasm
Periplasmic protein TorT	P38683	*Escherichia coli* (strain K12)	Periplasmic space
DNA-directed RNA polymerase subunit alpha	P0A7Z4	*Escherichia coli* (strain K12)	Cytoplasm
Putative acyl-CoA dehydrogenase AidB	P33224	*Escherichia coli* (strain K12)	Cytoplasm
Antitoxin VapB2	O07227	*Mycobacterium tuberculosis* (strain ATCC 25618 / H37Rv)	Unknown
HTH-type transcriptional regulator BetI	B7VQ27	*Vibrio atlanticus* (strain LGP32)	Cytoplasm
Catabolite control protein A	O07329	*Streptococcus mutans serotype c* (strain ATCC 700610 / UA159)	Cytoplasm

DNA-directed RNA polymerase subunit beta'	Q0BUQ5	*Granulibacter bethesdensis* (strain ATCC BAA-1260 / CGDNIH1)	Cytoplasm
<u>Antibiotics biosynthesis</u>			
Cytochrome P-450 monooxygenase DoxA	Q93MI2	*Streptomyces peucetius subsp. caesius*	Cytoplasm
<u>Nucleotide metabolism</u>			
Allantoate amidohydrolase	P77425	*Escherichia coli* (strain K12)	Cytoplasm
Bifunctional protein FolD	P75096	*Mycoplasma pneumoniae (strain ATCC 29342 / M129)*	Cytoplasm
Thymidylate kinase	Q6FZU8	*Bartonella quintana (strain Toulouse)*	Cytoplasm
dTTP/UTP pyrophosphatase	Q82ZA4	*Enterococcus faecalis* (strain ATCC 700802 / V583)	Cytoplasm
Phosphoribosylamine--glycine ligase	P15640	*Escherichia coli* (strain K12)	Cytoplasm
Phosphotransferase RcsD	P39838	*Escherichia coli* (strain K12)	Plasma membrane
DNA translocase FtsK 2	Q8XWX9	*Ralstonia solanacearum (strain GMI1000)*	Plasma membrane
Dihydroorotase	P96081	*Thermus aquaticus*	Cytoplasm
<u>Pathogenesis</u>			
LPS-assembly protein LptD	P31554	*Escherichia coli* (strain K12)	Cell outer membrane
3-deoxy-D-manno-octulosonic acid kinase	Q9PBJ1	*Xylella fastidiosa (strain 9a5c)*	Plasma membrane
T-DNA border endonuclease VirD2	P18592	*Agrobacterium fabrum* (strain C58 / ATCC 33970)	Cytoplasm
<u>Cell wall organization and structural maintenance</u>			
D-alanyl-D-alanine dipeptidase	P77790	*Escherichia coli* (strain K12)	Cytoplasm

1,6-anhydro-N-acetylmuramyl-L-alanine amidase AmpD	P13016	*Escherichia coli* (strain K12)	Cytoplasm
Membrane-bound lytic murein transglycosylase F	P0AGC5	*Escherichia coli* (strain K12)	Cell outer membrane
Undecaprenyl-diphosphatase	G0FS62	*Amycolatopsis mediterranei* (strain S699)	Plasma membrane
Lipid metabolism			
Acyl carrier protein phosphodiesterase	B1XEZ2	*Escherichia coli* (strain K12)	Cytoplasm
Crotonobetainyl-CoA reductase	P60584	*Escherichia coli* (strain K12)	Cytoplasm
Bifunctional polymyxin resistance protein ArnA	P77398	*Escherichia coli* (strain K12)	Protein containing complex
Long-chain-fatty-acid--CoA ligase	P69451	*Escherichia coli* (strain K12)	Cytoplasm
Medium/long-chain-fatty-acid--[acyl-carrier-protein] ligase MbtM	P0A4X9	*Mycobacterium bovis* (strain ATCC BAA-935 / AF2122/97)	Plasma membrane
Phosphatidylglycerol--prolipoprotein diacylglyceryl transferase	Q17X31	*Helicobacter acinonychis* (strain Sheeba)	Plasma membrane
Unknown			
Putative lipoprotein YfiB	P07021	*Escherichia coli* (strain K12)	Plasma membrane
Putative aminopeptidase FrvX	P32153	*Escherichia coli* (strain K12)	Cytoplasm
Probable ATP-binding protein YheS	P63389	*Escherichia coli* (strain K12)	Cytoplasm
Ureidoglycolate dehydrogenase (NAD(+))	W1F386	*Escherichia coli* (strain K12)	Cytoplasm
Inner membrane protein YbjJ	P75810	*Escherichia coli* (strain K12)	Plasma membrane
Probable lipoprotein YiaD	P37665	*Escherichia coli* (strain K12)	Plasma membrane
tRNA-dihydrouridine(20/20a) synthase	P32695	*Escherichia coli* (strain K12)	Cytoplasm
Putative phosphatase MPN_427	P75360	*Mycoplasma pneumoniae* (strain ATCC 29342 / M129)	Cytoplasm
Putative MgpC-like protein MPN_092	P75600	*Mycoplasma pneumoniae* (strain ATCC 29342 / M129)	Unknown
Probable ATP-dependent RNA helicase MG308 homolog	P75335	*Mycoplasma pneumoniae* (strain ATCC 29342 / M129)	Cytoplasm
Phosphoenolpyruvate-protein phosphotransferase	P75168	*Mycoplasma pneumoniae* (strain ATCC 29342 / M129)	Cytoplasm

Putative esterase/lipase 1	P75333	*Mycoplasma pneumoniae* (strain ATCC 29342 / M129)	Unknown
ATP synthase subunit alpha	Q50329	*Mycoplasma pneumoniae* (strain ATCC 29342 / M129)	Plasma membrane
Acyl carrier protein homolog	P75378	*Mycoplasma pneumoniae* (strain ATCC 29342 / M129)	Plasma membrane
Protein MPN_376	P75405	*Mycoplasma pneumoniae* (strain ATCC 29342 / M129)	Cytoplasm
UPF0597 protein DP0591	Q6AQQ3	*Desulfotalea psychrophila* (strain LSv54 / DSM 12343)	Cytoplasm
L-lactate oxidase	C0XIJ3	*Lentilactobacillus hilgardii* (strain ATCC 8290)	Cytoplasm
Flagellin	P06175	*Salmonella rubislaw*	Extracellular
5-methylthioadenosine/S-adenosylhomocysteine deaminase	A4J675	*Desulfotomaculum reducens* (strain MI-1)	Cytoplasm
Surface layer protein	P38538	*Brevibacillus choshinensis*	Extracellular
ESX-5 secretion system protein EccA5	P63745	*Mycobacterium bovis* (strain ATCC BAA-935 / AF2122/97)	Cytoplasm
Protein adenylyltransferase SelO	Q1H0D2	*Methylobacillus flagellatus* (strain KT / ATCC 51484 / DSM 6875)	Cytoplasm
Sugar fermentation stimulation protein homolog	A6W2V7	*Marinomonas sp.* (strain MWYL1)	Cytoplasm
Phase 2 flagellin	P52615	*Salmonella abortus-equi*	Extracellular
Putative membrane protein insertion efficiency factor	Q11JM2	*Chelativorans sp.* (strain BNC1)	Plasma membrane
UPF0358 protein BH2626	Q9K9L9	*Alkalihalobacillus halodurans* (strain ATCC BAA-125 / DSM 18197 / FERM 7344 / JCM 9153 / C-125)	Cytoplasm
UPF0114 protein PC1_0431	C6DJS4	*Pectobacterium carotovorum subsp. carotovorum* (strain PC1)	Plasma membrane
D-threo-3-hydroxyaspartate dehydratase	B2DFG5	*Delftia sp.* (strain HT23)	Cytoplasm
Methionine import ATP-binding protein MetN 1	Q63H29	*Bacillus cereus (strain ZK / E33L)*	Plasma membrane
FHA domain-containing protein FhaB	A0QNG6	*Mycolicibacterium smegmatis* (strain ATCC 700084 / mc(2)155)	Plasma membrane
